# Ferroptosis-related gene analysis revealing novel biomarkers and therapeutic targets in diffuse large B-cell lymphoma

**DOI:** 10.1038/s41598-025-22478-1

**Published:** 2025-11-04

**Authors:** Lushe Liu, Liqun Guo, Huiyang Zhang, Runhong Yu, Yuanyuan Hao, Xiaoyan Dong, Rui Dou, Zunmin Zhu, Linna Cheng

**Affiliations:** 1 Institute of Hematology, Henan Key Laboratory of Stem Cell Clinical Application and Key Technology, Henan Provincial People’s Hospital, Zhengzhou University People’s Hospital, Henan University People’s Hospital, Zhengzhou, 450003 Henan China; 2 Department of Hematology, Henan Provincial People’s Hospital, Zhengzhou University People’s Hospital, Henan University People’s Hospital, Zhengzhou, 450003 Henan China

**Keywords:** Diffuse large b-cell lymphoma, Ferroptosis, Key genes, Prognostic model, Cancer, Biomarkers, Diseases, Oncology

## Abstract

Diffuse large B-cell lymphoma (DLBCL) is an aggressive, heterogeneous non-Hodgkin lymphoma with high relapse rates and drug resistance, which necessitates novel biomarkers. This study aimed to fill a research gap by investigating the function of ferroptosis—an iron-dependent form of programmed cell death—in DLBCL, an area that remains inadequately explored. Employing a comprehensive bioinformatics framework, we analyzed ferroptosis-related genes in The Cancer Genome Atlas-DLBCL dataset using consensus clustering and differential expression, somatic mutation, copy number variation (CNV), gene ontology and pathway enrichment, and immune infiltration analyses. Our analysis identified two DLBCL subtypes, revealing 912 differentially expressed genes, including 24 ferroptosis-related differentially expressed genes (FRDEGs). Enrichment analyses indicated that these genes are involved in crucial biological pathways, including the lipoxygenase pathway and inflammatory regulation, while immune infiltration assessment highlighted significant correlations with specific immune cell types, particularly the positive correlation of *IDO1* with M1 macrophages and the negative correlation of *IFNG* with memory B cells. Further, we established a prognostic risk model incorporating *CDKN1A*, *KLF2*, and *IFNG* that holds promise for predicting patient outcomes. These findings demonstrate that ferroptosis regulates DLBCL progression and identify potential biomarkers/therapeutic targets requiring validation to develop new therapies.

**Supplementary Information:**

The online version contains supplementary material available at 10.1038/s41598-025-22478-1.

## Introduction

Diffuse large B-cell lymphoma (DLBCL) is a clinically challenging subtype of non-Hodgkin lymphoma characterized by high recurrence rates and poor prognoses. Standard treatment protocols such as R-CHOP immunochemotherapy (rituximab, cyclophosphamide, doxorubicin, vincristine, prednisone) can successfully cure 60‒70% of patients with DLBCL. However, a substantial proportion of patients who do not achieve remission encounter resistance to treatment, leading to unfavorable clinical results^1,2^. DLBCL exhibits notable genetic and clinical heterogeneity, which impacts its diagnosis, prognosis, and treatment strategies^3,4^. Gene expression profiling and high-throughput sequencing have enabled the systematic detection of the distinct molecular signatures and genetic causes of DLBCL subtypes; however, its heterogeneity necessitates a more refined molecular classification to enhance treatment efficacy and patient outcomes^4^. Consequently, elucidating the genetic foundation of this disease and exploring innovative therapeutic approaches are crucial.

Ferroptosis, a form of programmed necrosis, is characterized by iron dependence and reliance on reactive oxygen species to induce cell death. Previous studies have established the crucial role of ferroptosis in various cancers, wherein it influences tumor cell proliferation, apoptosis, and immune evasion^5^. Notably, among 117 unique cancer cell lines derived from various tissues, including hematopoietic and lymphoid tissues, colorectum, lung, ovary, and skin, cells from DLBCL exhibit particularly high sensitivities to ferroptosis inducers^6^. Current research establishes ferroptosis dysregulation as a prognostic biomarker in DLBCL progression^7^. Mechanistically, pharmacological induction of ferroptosis synergistically enhances therapeutic responses to chemo-radiotherapy^7,8^. Therefore, ferroptosis has emerged as a promising prognostic biomarker and a viable therapeutic target for DLBCL^9^. Increased iron uptake and accumulation in DLBCL cells promote cell growth and survival, leading to poorer patient outcomes^7,10^. In contrast, the accumulation of intracellular ferrous iron and the suppression of the expression of the anti-ferroptosis protein GPX4 enhances ferroptosis-mediated cell death and inhibits DLBCL growth^11^. This inconsistency emphasizes the complex dynamics of ferroptosis in DLBCL and reinforces the need for a deeper understanding of its fundamental mechanisms to formulate effective treatment approaches.

By identifying and characterizing ferroptosis-related differentially expressed genes (FRDEGs), this study aimed to identify novel molecular subtypes of DLBCL, thereby enhancing our understanding of its pathophysiology and potential therapeutic targets.

## Results

### Integrated analytical workflow

**Fig. 1:**
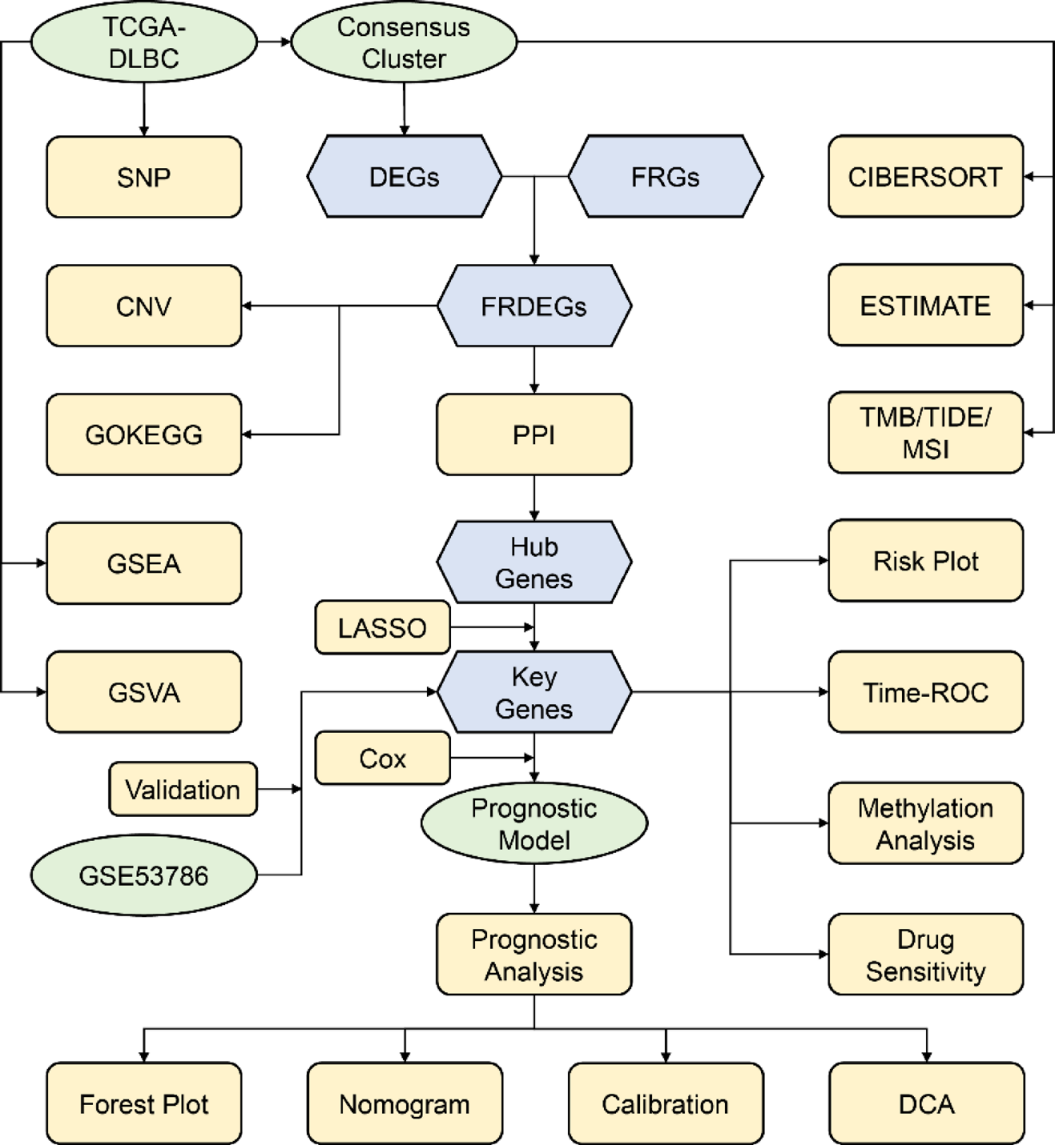
Flow chart for the comprehensive analysis of DLBC and ferroptosis. TCGA, The Cancer Genome Atlas; DLBC, Lymphoid Neoplasm Diffuse Large B-Cell Lymphoma༛DEGs, Differentially Expressed Genes༛FRGs, Ferroptosis-Related Genes༛FRDEGs, Ferroptosis-Related Differentially Expressed Genes༛SNP, Single Nucleotide Polymorphisms༛CNV, Copy Number Variations༛GO, Gene Ontology༛KEGG, Kyoto Encyclopedia of Genes and Genomes༛GSEA, Gene Set Enrichment Analysis༛GSVA, Gene Set Variation Analysis༛PPI, Protein-Protein Interaction༛TMB, Tumor Mutation Burden༛TIDE, Tumor Immune Dysfunction.

This study delineates a multi-omics framework (Fig. 1) to explore the expression characteristics and clinical significance of ferroptosis-related genes (FRGs) in DLBCL. Consensus clustering stratified The Cancer Genome Atlas (TCGA)-DLBCL into molecular subtypes (Cluster 1/2). Subsequent DESeq2-based screening identified differentially expressed genes (DEGs) intersecting with FerrDb-curated ferroptosis-related genes (FRGs), consequently yielding 24 FRDEGs. Following subtyping, somatic mutation (SM) landscapes were profiled in TCGA-DLBCL with quantification of mutation frequencies. Concurrently, GISTIC analysis mapped FRDEG-associated copy number variation (CNV), while highlighting recurrent amplifications/deletions. Functional enrichment analyses via Gene Ontology (GO) and Kyoto Encyclopedia of Genes and Genomes (KEGG) decoded FRDEG-linked biological processes, while Gene Set Enrichment Analysis (GSEA) and Gene Set Variation Analysis (GSVA) quantified pathway activity differences between clusters. Further, the tumor microenvironment was characterized through Immune infiltration quantification (CIBERSORT), FRDEG-immunocyte correlation analyses and ESTIMATE algorithm. Protein-protein interaction (PPI) networking refined 15 hub genes from FRDEGs. Additionally, LASSO regression established a 3-gene prognostic signature, stratifying patients into high/low-risk cohorts. The expression of these three key genes was validated using the GSE53786 dataset. The model’s clinical utility was validated via methylation dynamics, drug sensitivity, and decision curve analysis (DCA), thus bridging molecular taxonomy to therapeutic vulnerabilities.

## DLBCL-associated FRDEG analysis

**Fig. 2:**
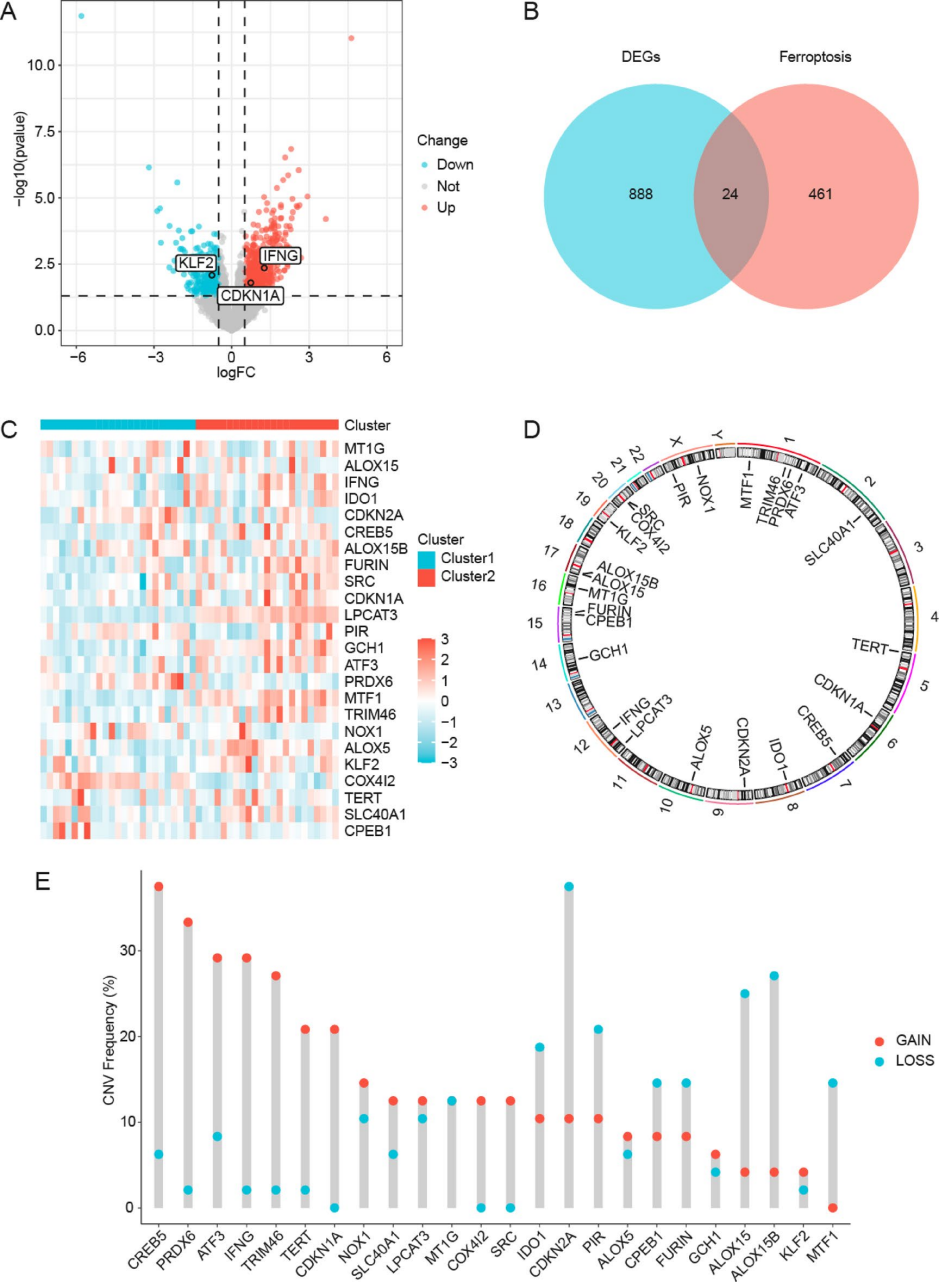
Multi-dimensional Characterization of Ferroptosis-Related Transcriptional Alterations in DLBCL. **A**. Volcano plot of DEG analysis for Clusters 1 and 2 in TCGA-DLBCL dataset. Red, blue, and gray dots represent upregulated DEGs, downregulated DEGs, and insignificant genes, respectively. **B**. Intersectional Venn diagram of DEGs and FRGs. **C**. FRDEG expression heatmap. Blue and red samples represent Clusters 1 and 2, respectively. Heatmap color indicates the expression level; darker red and blue colors signify higher and lower expression levels, respectively. **D**. Chromosomal mapping of the FRDEGs. **E**. CNVs of FRDEGs are presented in TCGA-DLBCL. TCGA, The Cancer Genome Atlas; DLBCL, diffuse large B-cell lymphoma; DEG, differentially expressed gene; FRDEG, ferroptosis-related differentially expressed gene; CNV, copy number variation.

Consensus clustering analysis successfully delineated two unique DLBCL subtypes: Clusters 1 (25 samples) and 2 (23 samples; Fig. S1). Notably, 912 genes met the |log_2_FC| > 0.5 and *p* < 0.05 thresholds, including 663 upregulated genes and 249 downregulated genes (Fig. 2A). Given that ferroptosis results from the balance between promoting and inhibiting factors, we intersected all 912 DEGs with FRGs and identified 24 FRDEGs (Fig. 2B): *MT1G*,* ALOX15*,* IFNG*,* IDO1*,* CDKN2A*,* CREB5*,* ALOX15B*,* FURIN*,* SRC*,* CDKN1A*,* LPCAT3*,* PIR*,* GCH1*,* ATF3*,* PRDX6*,* MTF1*,* TRIM46*,* NOX1*,* ALOX5*,* KLF2*,* COX4I2*,* TERT*,* SLC40A1*,* and CPEB1*. The aforementioned 24 FRDEGs represent proposed core mediators linking ferroptosis to DLBCL pathogenesis. Their expression patterns hold potential as molecular classifiers for disease subtyping, while subsequent functional validation would require confirming their roles in ferroptosis regulation and tumor microenvironment reprogramming. This framework could provide a molecular basis for refined DLBCL stratification.

We examined the expression variances of FRDEGs across various sample cohorts within TCGA-DLBCL dataset (Fig. 2C). Subsequently, the chromosomal locations of the 24 FRDEGs were examined, which resulted in the creation of a chromosomal localization map (Fig. 2D). Chromosome mapping revealed high FRDEG concentration on chromosome 1, including *PRDX6*, *ATF3*, *TRIM46*, and *MTF1*.

We performed CNV analysis on the TCGA-DLBC cohort using GISTIC2.0, thereby identifying amplification (Fig. S2A) and deletion (Fig. S2B) hotspots. Notably, most of FRDEGs exhibited significant CNV events, with copy number gains (e.g., *CREB5*) and losses (e.g., *CDKN2A*) showing distinct chromosomal clustering (Fig. 2E). Critically, CNV directionality showed a strong correlation with transcriptomic changes: amplified *CREB5* showed significant upregulation specifically in Cluster 2, while deleted *CDKN2A* was downregulated in Cluster 2 (Fig. 2C). These findings support CNV-driven dosage effects as a key regulatory mechanism modulating ferroptosis pathway activity in DLBCL subtypes. Previous studies have demonstrated that CREB5 amplification likely promotes tumor cell survival by activating the MAPK/ERK signaling pathway^12^, while CDKN2A deletion correlates with chemoresistance in DLBCL patients^13^. These CNV events may modulate the expression of ferroptosis-related regulators (e.g., GPX4 or ACSL4), consequently influencing oxidative stress tolerance in DLBCL.

Analysis of SM in the TCGA-DLBCL cohort identified nine predominant categories, with missense mutations representing the most frequent variant type. Meanwhile, single nucleotide polymorphisms, particularly C-to-T transitions, constituted the principal mutation class (Fig. S2C). Subsequently, we examined SM status and ranked the 30 genes exhibiting the highest mutation frequencies for enhanced visualization (Fig. S2D), with the *BTG2 gene* exhibiting the highest mutation frequency (28%). In the subsequent analysis, the mutation status of each sample in TCGA-DLBCL dataset proved that C-to-T transitions were the most prevalent mutations (Fig. S2E). Further, correlation analysis was performed on the top 20 genes ranked by mutation frequency, which resulted in the creation of a correlation heatmap (Fig. S2F).

## Functional enrichment analysis of FRDEGs in DLBCL

**Fig. 3:**
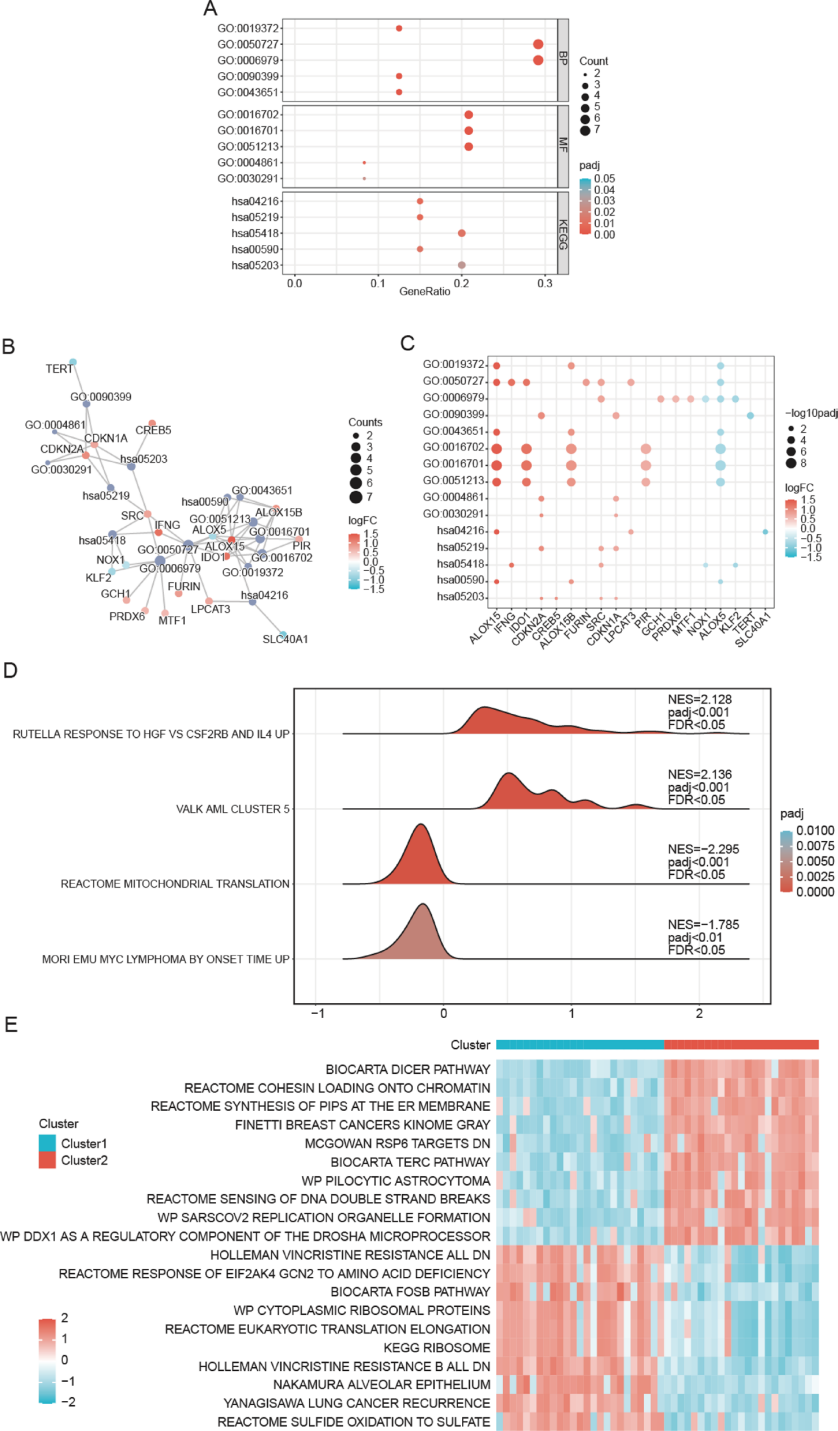
Functional Annotation of Ferroptosis Regulatory Networks in DLBCL. **A**. Bubble plot displaying the results of GO and KEGG enrichment analysis of FRDEGs. The abscissa is GeneRatio, while the ordinate is GO and KEGG terms. Bubble color indicates the adj.p size. **B**. Network diagram of GO and KEGG enrichment analysis results of FRDEGs. Purple nodes indicate entries, red or blue nodes indicate FRDEGs, and color indicates log_2_FC value. The lines represent the relationship between the entries and molecules. **C**. Heatmap of the results of GO and KEGG enrichment analysis of FRDEGs. Circles indicate that the corresponding FRDEGs were enriched under the corresponding entries. The circle color indicates the log_2_FC value. Circle size indicates the adj.p value. The screening criteria for GO and KEGG enrichment analysis were adj.*p* < 0.05 and FDR value (q value) < 0.25. The p correction method was Benjamini–Hochberg (BH). **D**. Landscape visualization of GSEA in TCGA-DLBCL. Each mountain’s position relative to zero encodes NES direction (left = negative, right = positive). The area of a mountain indicates the absolute value of NES; The color of the mountain indicates the size of the adj.p value. **E**. Heatmap of enrichment scores between Clusters 1 and 2 of GSVA results in TCGA-DLBCL dataset. Blue and red samples represent Clusters 1 and 2, respectively; the Heatmap indicates the enrichment score. GO, Gene Ontology; KEGG, Kyoto Encyclopedia of Genes and Genomes; FRDEG, ferroptosis-related differentially expressed gene; FDR, false discovery rate; GSEA, gene set enrichment analysis; GSVA, gene set variation analysis.

GO and KEGG enrichment analyses for FRDEGs revealed that these FRDEGs were predominantly associated with pathways linked to lipoxygenase activity, inflammatory response regulation, oxidative stress responses, and gene expression processes (Table S1). Furthermore, these FRDEGs exhibited significant enrichment in several molecular functions including oxidoreductase activity, dioxygenase activity, and inhibition of cyclin-dependent protein serine/threonine kinase activity (Fig. 3A). Building on these functional annotations, we integrated the log_2_FC values of the FRDEGs to construct a comprehensive interaction network and enrichment heatmap (Fig. 3B‒C). Critically, key FRDEGs such as ALOX15 and ALOX5 regulated lipid peroxidation to mediate ferroptosis susceptibility, thereby offering actionable targets for combination chemotherapy regimens.

The synergistic application of GSEA and GSVA provided complementary dimensionality to characterize ferroptosis pathway dysregulation in DLBCL subtypes. GSEA methodology (pre-ranked genes by log₂FC) revealed global pathway activation patterns in TCGA-DLBCL (FDR < 0.25; Table S2), identifying ferroptosis-related pathways with coordinated enrichment between Cluster 1 and Cluster 2, including the rutella response to hepatocyte growth factor vs. colony-stimulating factor 2 receptor subunit beta and interleukin-4 up, VALK AML CLUSTER 5, mitochondrial translation, and Eµ-Myc lymphoma progression by onset time (Fig. 3D). Complementary GSVA assessment of the same cohort (raw expression input) quantified subtype-specific pathway activities (Table S3), confirming significant variation in top enriched pathways (adj. *p* < 0.05) between clusters (Fig. 3E). Key heterogeneous pathways included the biocarta dicer pathway, cohesin loading onto chromatin, and phosphatidylinositol phosphate synthesis at the endoplasmic reticulum membrane.

## Immune infiltration and tumor microenvironment characteristics in TCGA-DLBCL subgroups

**Fig. 4:**
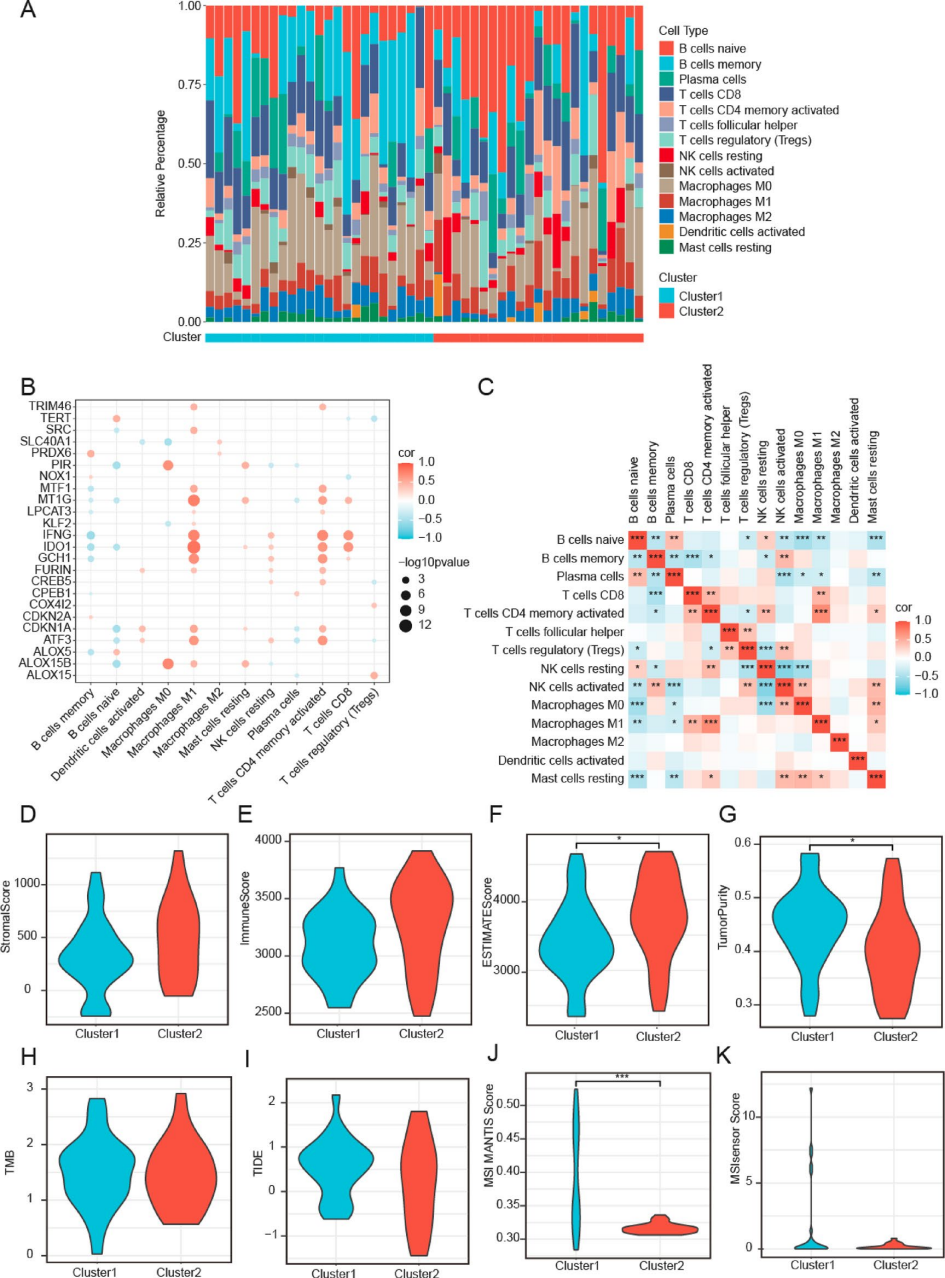
Immune cell infiltration and ferroptosis-related gene correlation in DLBCL. **A**. Bar chart depicting immune cell proportion in TCGA-DLBCL dataset. The x-axis represents individual samples, with blue and red samples belonging to Clusters 1 and 2, respectively. The y-axis shows the percentage of immune cell infiltration abundance, with different colors representing different immune cell types. **B**. Bubble plot illustrating the correlation between FRDEGs and immune cell infiltration abundance. Colors indicate correlation direction: red for positive and blue for negative. Color intensity reflects correlation magnitude. Circle size corresponds to the significance level. **C**. Heatmap showing the correlation between immune cell infiltration abundances. Colors represent correlation direction and strength: red for positive and blue for negative. **D–G**. Comparison of stromal score (**D**), immune score (**E**), ESTIMATE score (**F**), and tumor purity (**G**) between Clusters 1 (blue) and 2 (red) in the dataset. Violin plots are used to visualize score distribution between both clusters.** H–K**. Comparison of TMB (**H**), TIDE score (**I**), MSI MANTIS (**J**), and MSI sensor (**K**) between Clusters 1 (blue) and 2 (red) in the dataset. Violin plots show the distributions of these metrics. * *p* < 0.05, *** *p* < 0.001. TCGA, The Cancer Genome Atlas; DLBCL, diffuse large B-cell lymphoma; FRDEG, ferroptosis-related differentially expressed gene; TMB, Tumor Mutation Burden; TIDE, Tumor Immune Dysfunction and Exclusion; MSI, Micro-satellite Instability.

The CIBERSORT algorithm was employed to assess the distribution of immune cell infiltration across two TCGA-DLBCL clusters (Fig. 4A). Examination of the associations between FRDEGs and immune cell infiltration (Fig. 4B) showed that *IDO1* demonstrated the highest positive correlation with M1 macrophages (*r* = 0.826, *p* < 0.001), whereas *IFNG* demonstrated the most pronounced negative correlation with memory B cells (*r* = −0.560, *p* < 0.001). Furthermore, correlation analysis of the abundance of immune cell infiltration (Fig. 4C) highlighted a robust positive correlation between activated CD4^+^ memory T cells and M1 macrophages (*r* = 0.704, *p* < 0.001), whereas the most significant negative correlation was noted between resting and activated natural killer (NK) cells (*r* = −0.669, *p* < 0.001). The higher expression of *IDO1* in Cluster 2 (Fig. 2C) may indicate an increased infiltration of activated CD4^+^ memory T cells and M1 macrophages in this cluster. These findings collectively indicate that the 24 FRDEGs (e.g., *IDO1*,* IFNG*) could serve as supplementary biomarkers for characterizing tumor microenvironment status, offering valuable insights for predicting immunotherapeutic responses.

Upon computing the stromal scores (Fig. 4D), immune scores (Fig. 4E), estimate scores (Fig. 4F), and tumor purity (Fig. 4G) for patients in the two TCGA-DLBCL clusters, we found that both the estimate score and tumor purity exhibited significant differences between clusters (*p* < 0.05). Specifically, Cluster 2 presented a higher estimate score than Cluster 1 (Fig. 4F), whereas Cluster 1 demonstrated greater tumor purity than Cluster 2 (Fig. 4G). We further assessed the variations in tumor mutation burden (TMB, Fig. 4H), tumor immune dysfunction and exclusion (TIDE, Fig. 4I), and Microsatellite Instability (MSI) scores (Fig. 4J-K). While TMB and TIDE showed comparable distributions, Cluster 1 had a higher MSI MANTIS score than Cluster 2 (*p* < 0.05, Fig. 4K).

## Identification and prognostic value of key FRDEGs in DLBCL

We conducted a PPI analysis using the STRING database, which enabled us to establish a network comprising 24 FRDEGs (Fig. S3A). Subsequently, we employed the Density of Maximum Neighborhood Component (DMNC) algorithm via the CytoHubba plugin in Cytoscape and identified the top 15 hub genes that are anticipated to be pivotal in ferroptosis and related biological functions. These hub genes were *TERT*,* ALOX15*,* IDO1*,* SRC*,* ALOX15B*,* NOX1*,* ALOX5*,* MT1G*,* CDKN1A*,* CDKN2A*,* KLF2*,* ATF3*,* FURIN*,* LPCAT3*, and *IFNG* (Fig. S3B). Finally, we used the GSCA platform to examine the expression profiles of hub genes across various cancer types (Additional Fig. S3C), thereby providing critical insights into candidate biomarkers and therapeutic targets for various malignancies. For visualization, we illustrated the LASSO regression model (Additional Fig. S3D) and mapped the trajectories of variables (Additional Fig. S3E). The genes selected through this LASSO regression analysis are referred to as key genes: *CDKN1A*,* KLF2*, and *IFNG*. To calculate the risk score for each specimen, we used the following formula:

$$\:\:Risk\:Score\:=\:0.3320\:\ast\:\:CDKN1A\:+\:0.1560\:\ast\:\:KLF2\:+\:0.0996\:\ast\:\:IFNG$$

**Fig. 5:**
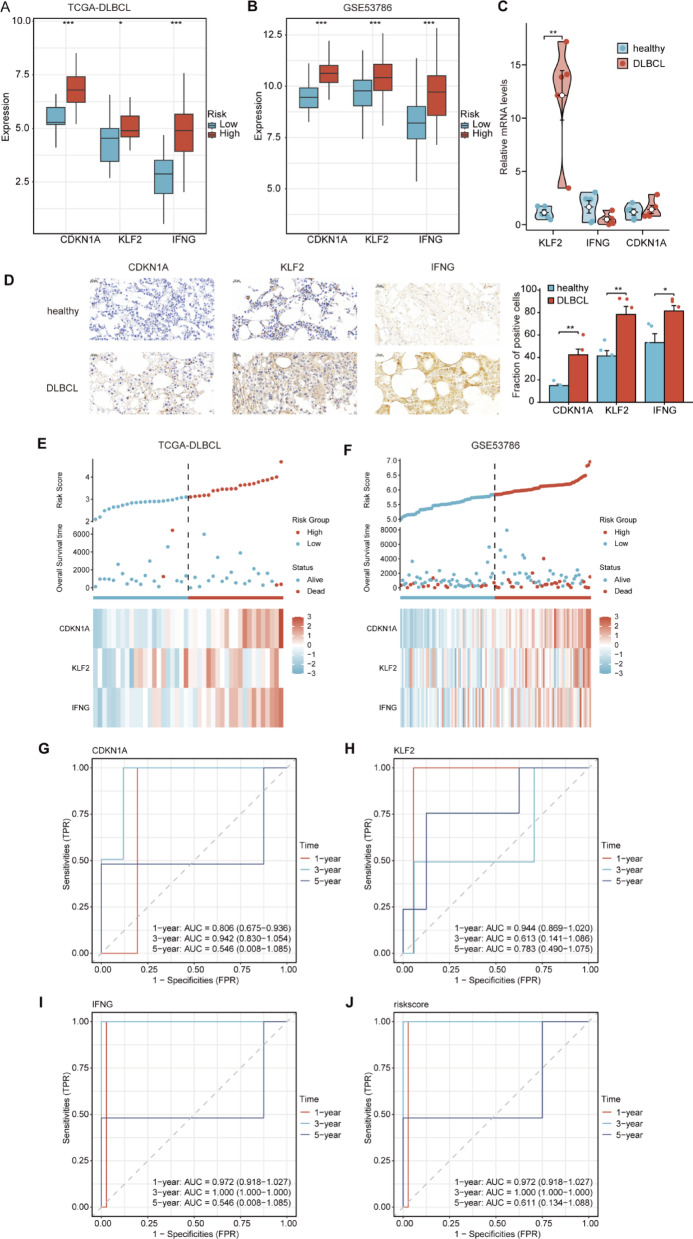
Expression Profiles and Prognostic Value of Key Genes in DLBCL. **A**–**B**. Expression profiles of key genes in TCGA-DLBCL (**A**) and GSE53786 (**B**) datasets. The x-axis represents key genes, and the y-axis represents gene expression levels. Blue and red samples represent the low- and high-risk groups, respectively. **C**. mRNA levels of key genes in DLBCL and healthy samples from an in-house dataset. **D**. Immunohistochemical analysis of CDKN1A, KLF2 and IFNG expression in DLBCL and healthy samples from an in-house dataset.. Immunohistochemical analysis of CDKN1A, KLF2 and IFNG expression in DLBCL and healthy samples from an in-house dataset. **E–F**. Risk factor plots generated by the LASSO regression model for TCGA-DLBCL (**E**) and GSE53786 (**F**) datasets. The x-axis represents the risk score, and the y-axis represents the expression levels of the selected genes. The vertical dashed line indicates the median risk score, dividing the samples into high- and low-risk groups. **G–J**. Time-dependent ROC curves for the key genes *CDKN1A* (**G**), *KLF2* (**H**), and *IFNG* (**I**) and the risk score (**J**) in TCGA-DLBCL dataset at 1-, 3-, and 5- years. The x-axis represents the false positive rate (FPR), and the y-axis represents the true positive rate (TPR). *p < 0.05, **p < 0.01, ***p < 0.001. TCGA, The Cancer Genome Atlas; DLBCL, diffuse large B-cell lymphoma; LASSO, Least Absolute Shrinkage and Selection Operator; ROC, receiver operating characteristic; TPR, True Positive Rate; FPR, False Positive Rate.

Based on the median risk score generated by the prognostic model, DLBCL samples were categorized into high- and low-risk groups. To examine the expression patterns of key genes in DLBCL samples, we assessed the expression of *CDKN1A*, *KLF2*, and *IFNG* across the high- and low-risk groups based on TCGA-DLBCL (Fig. 5A) and GSE53786 datasets (Fig. 5B). Notably, the expression of these three key genes significantly increased in the high-risk group compared to the low-risk group across both TCGA-DLBCL and GSE53786 datasets (*p* < 0.05). We extended our investigation by collecting peripheral blood and bone marrow samples from five patients with DLBCL and five normal individuals to analyze the expression of these key genes in a clinical setting. Quantitative reverse-transcription polymerase chain reaction (qRT-PCR) analysis of peripheral blood revealed significantly elevated *KLF2* mRNA in DLBCL compared to controls, while *CDKN1A* and *IFNG* transcripts showed no significant difference (Fig. 5C). In contrast, immunohistochemistry analysis of bone marrow samples demonstrated significantly increased protein levels of KLF2, CDKN1A, and IFNG in DLBCL (Fig. 5D). The discordance between mRNA and protein levels of CDKN1A and IFNG suggested that their upregulation may be mediated by post-transcriptional mechanisms (e.g., enhanced translation or protein stability) rather than transcriptional activation. This observation could also reflect tissue-specific microenvironments between peripheral blood and bone marrow. Although limited sample size may contribute to statistical variability, the elevated protein levels of all three biomarkers indicate their potential functional significance in DLBCL pathogenesis.

To enhance the prognostic significance evaluation of CDKN1A/KLF2/IFNG in DLBCL, risk factor maps were generated (Fig. 5E‒F). The notable disparities in expression patterns between the high- and low-risk cohorts indicate that these key genes might act as effective biomarkers for forecasting patient outcomes and providing individualized treatment approaches for DLBCL. Furthermore, time-dependent receiver operating characteristic (ROC) curves were generated for TCGA-DLBCL (Fig. 5G‒J) and GSE53786 (Fig. S4) datasets. Notably, the tri-gene signature exhibited exceptional short-term prognostic accuracy in the TCGA cohort (1- and 3-year AUCs > 0.97), while maintaining modest but statistically significant discriminatory power in the independent validation cohort (*p* < 0.05 across all timepoints). This establishes CDKN1A/KLF2/IFNG as a clinically relevant stratification tool for DLBCL progression risk, with particular utility for near-term prognosis.

Given the demonstrated prognostic value of these key genes, we further investigated potential underlying regulatory mechanisms by examining their methylation profiles in the TCGA cohort. Methylation profiling of three key genes in the TCGA cohort (Figs. S5A-C) revealed annotated deltaBeta distributions across loci (Fig. S5A) and comparable Beta values between risk groups (Fig. S5B), with no significant differential methylation at 42 interrogated loci. Subsequent integrative analysis of gene expression and methylation beta values identified locus-specific regulatory relationships (Fig. S5C). Specifically, although the methylation loci of these key genes did not differ significantly between risk groups, methylation levels at specific loci demonstrated significant correlations with corresponding gene expression levels (Fig. S5C). This finding suggests that epigenetic modifications at these regulatory sites may influence gene expression through transcriptional control mechanisms.

### Analysis of drug sensitivity in patients with High- and Low-Risk DLBCL

**Fig. 6:**
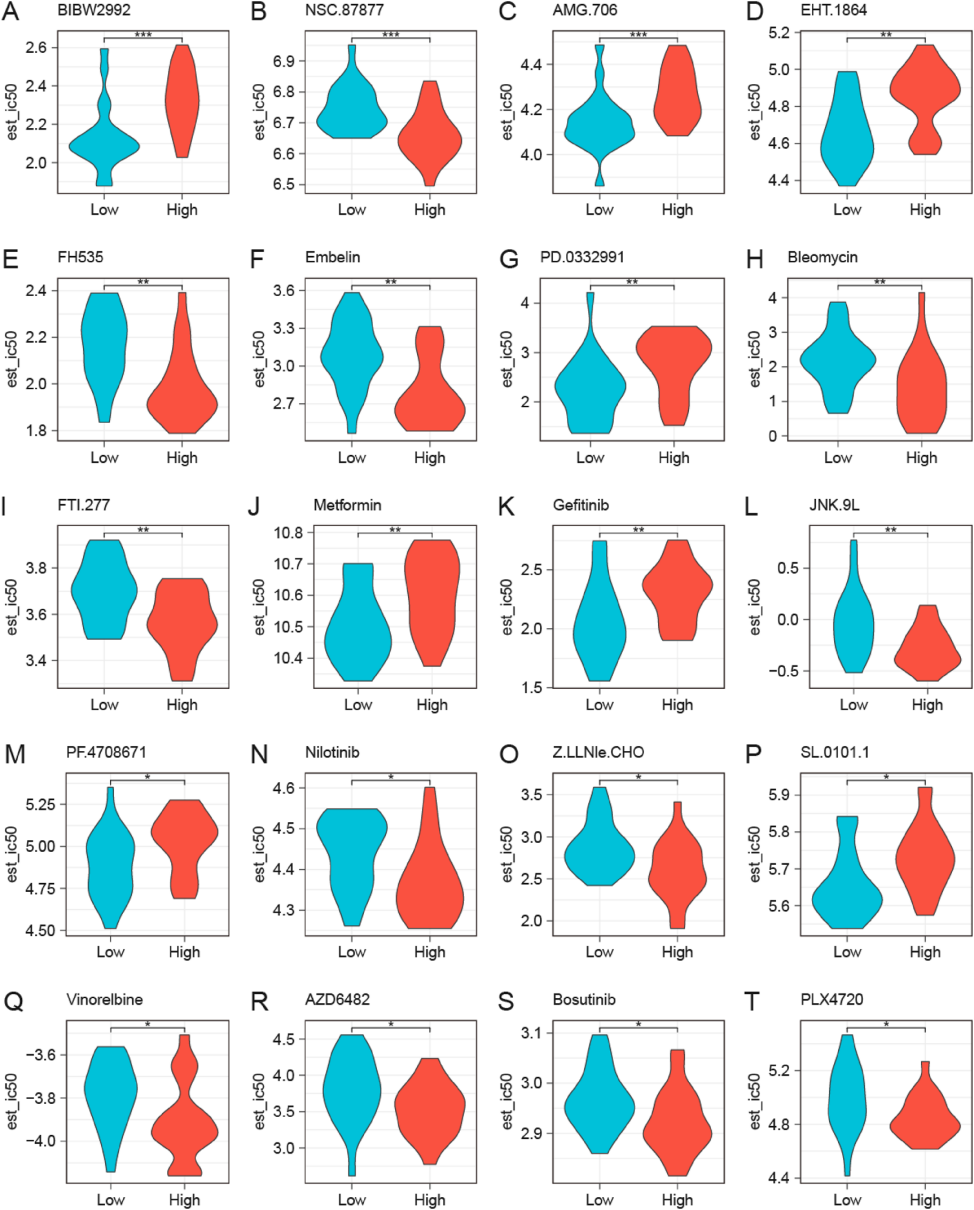
Drug Sensitivity Analysis of DLBCL. **A**–**T**. Drug sensitivity analysis of the high- and low-risk DLBCL groups from TCGA-DLBCL dataset, based on GDSC database. Blue samples represent the low-risk group, and red samples represent the high-risk group. * *p* < 0.05, ** *p* < 0.01; *** *p* < 0.001. TCGA, The Cancer Genome Atlas; DLBCL, diffuse large B-cell lymphoma; GDSC, Genomics of Drug Sensitivity in Cancer.

We utilized drug sensitivity information obtained from the Genomics of Drug Sensitivity in Cancer (GDSC) database as the training set to predict DLBCL responsiveness to various commonly used anticancer agents and assessed variations in drug sensitivity using TCGA-DLBCL dataset. Beyond statistical significance (*p* < 0.001), we prioritized drugs with established biological relevance to DLBCL pathogenesis. The top 20 candidates included BIBW2992 (afatinib), NSC.87,877 (paclitaxel), and AMG.706 (motesanib) (Fig. 6). Specifically, high-risk subgroup exhibited marked sensitivity to NSC.87,877 (paclitaxel), FH535, Ebelin, bleomycin, FTI.277 (Farnesyltransferase Inhibitor), JNK.9 L (JNK inhibitor), nilotinib, and Z.LLNle.CHO (*p* < 0.001), suggesting their potential as targeted therapeutics for chemoresistant DLBCL. Conversely, low-risk cases demonstrated responsiveness to EGFR/PI3K inhibitors (e.g., BIBW2992), AMG-706, EHT-1864, metformin, gefitinib, PF.4,708,671, and SL.0101.1. This aligns with aberrant PI3K/AKT activation in molecular subsets like germinal center B-cell-like (GCB)-DLBCL^14^, indicating distinct vulnerability profiles tied to risk stratification.

## Development of a prognostic risk model for DLBCL

**Fig. 7:**
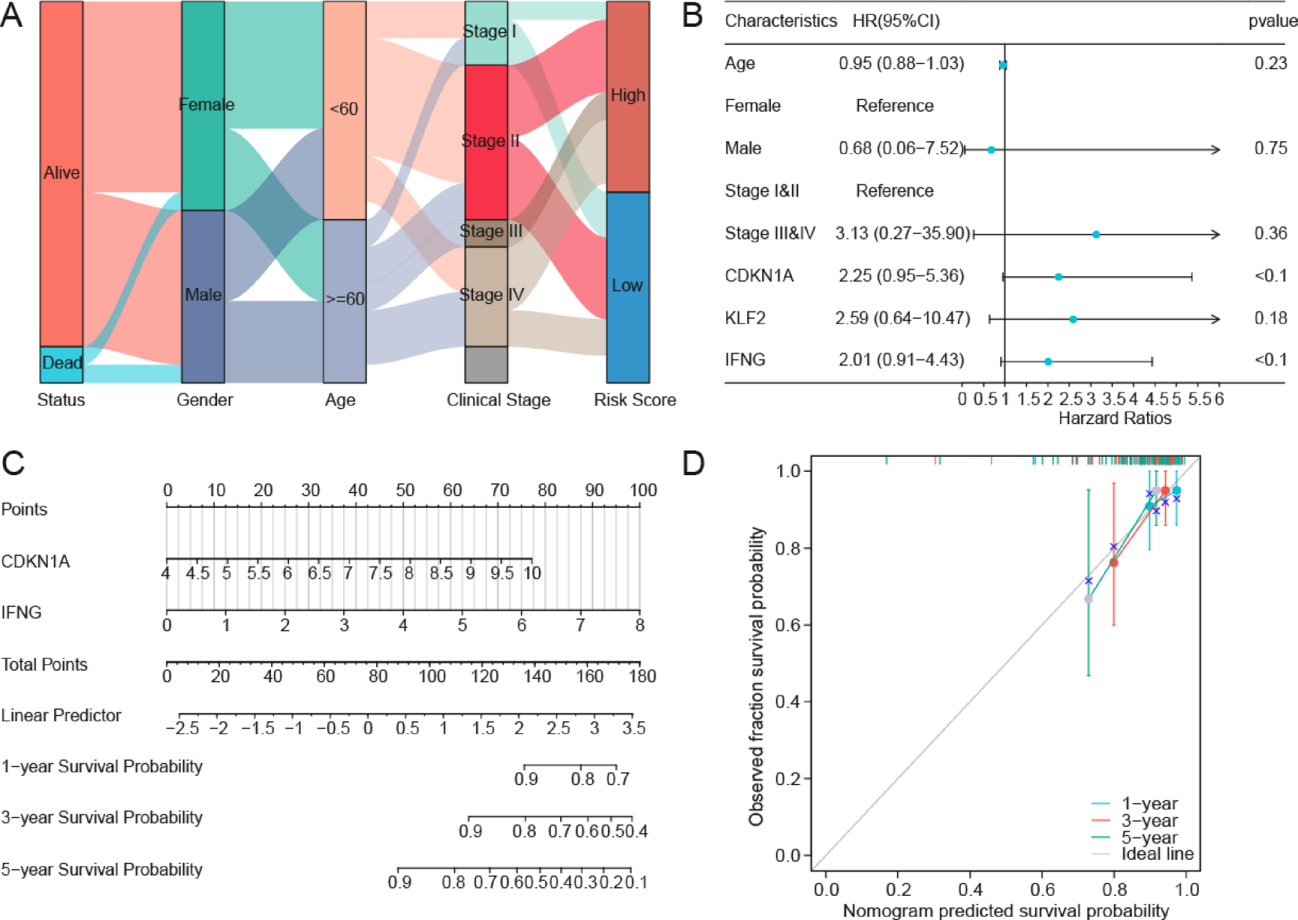
Cox Regression Analysis of DLBCL. **A**. Sankey diagram of clinical pathological characteristics in TCGA-DLBCL dataset. **B**. Forest plot of univariate Cox regression analysis for clinical information and three key genes. **C**–**D**. Prognostic nomogram (**C**) and calibration curve (**D**) from Multivariate Cox Regression Model. TCGA, The Cancer Genome Atlas; DLBCL, diffuse large B-cell lymphoma.

Baseline characteristics demonstrating significant correlations between risk stratification and clinicopathological variables are summarized in Table 1, with dynamic inter-variable relationships visualized through a Sankey diagram (Fig. 7A). Clinical data from TCGA-DLBCL, along with pivotal genes, were subjected to univariate Cox regression analysis (Fig. 7B). Subsequently, all variables demonstrating a *p* < 0.1 in the univariate analysis were included in the multivariate Cox regression model. This approach enabled the development of a nomogram illustrating the relationships among these variables (Fig. 7C). The Multivariate Cox regression analysis identified *CDKN1A* and *IFNG* as considerable prognostic factors. Comprehensive results of both the univariate and multivariate Cox regression analyses were presented in Table 2. Furthermore, the DLBCL prognostic risk model demonstrated robust temporal calibration accuracy, with 1-, 3-, and 5-year survival outcomes closely matching the diagonal line of the ideal model (Fig. 7D), thereby validating the accuracy of the prognostic model.

**Table 1:** Baseline table of TCGA-DLBCL grouped by risk Score.

Characteristics	Group	High (*N* = 21)	Low (*N* = 21)	*p*
Gender	Female	12 (57.1%)	11 (52.4%)	1
	Male	9 (42.9%)	10 (47.6%)	
Age	Mean ± SD	54.8 ± 15.8	57.0 ± 13.5	0.631
Clinical Stage	Stage I	4 (22.2%)	3 (15%)	0.803
	Stage II	7 (38.9%)	10 (50%)	
	Stage III	2 (11.1%)	1 (5%)	
	Stage IV	5 (27.8%)	6 (30%)	

TCGA, The Cancer Genome Atlas; DLBCL, Diffuse Large B-Cell Lymphoma; SD, standard deviation.

**Table 2:** Univariate and multivariate Cox regression analysis of TCGA-DLBCL.

Characteristics	Univariate HR (95% CI)	Univariate *p*	Multivariate HR (95% CI)	Multivariate *p*
Age	0.95 (0.88‒1.03)	0.23		
Gender				
Female	Reference			
Male	0.68 (0.06‒7.52)	0.75		
Clinical Stage				
Stage I&II	Reference			
Stage III&IV	3.13 (0.27‒35.90)	0.36		
CDKN1A	2.25 (0.95‒5.36)	0.07	1.56 (0.50‒4.86)	0.44
KLF2	2.59 (0.64‒10.47)	0.18		
IFNG	2.01 (0.91‒4.43)	0.09	1.55 (0.59‒4.07)	0.38

TCGA, The Cancer Genome Atlas; DLBCL, Diffuse Large B-Cell Lymphoma; CI, confidence interval.

DCA was employed to assess the clinical applicability of the prognostic risk model for DLBCL over 1-, 3-, and 5-years (Additional Fig. S6A-C). In DCA, a model line consistently exceeding both “All Positive” and “All Negative” within a designated interval signifies a more substantial net benefit and enhanced model performance. The findings demonstrated that the prognostic model offers considerable advantages over simplistic all-positive or all-negative predictions.

## Discussion

Our molecular stratification delineated two DLBCL subtypes. Cluster 2 showed significantly elevated ESTIMATE scores with enhanced infiltration of immunocytes (e.g., M1 macrophages, activated CD4^+^ memory T cells), mirroring the NF-κB-driven inflammatory microenvironment characteristic of the Activated B-cell-like (ABC) subtype^15^ In contrast, Cluster 1 exhibited higher tumor purity, which was consistent with the GCB subtype’s tumor cell-dominant phenotype^16^. The 24 FRDEGs exhibited significant expression differences between the two clusters. Notably, genes involved in inflammation regulation and ferroptosis, such as *ALOX15* and *IDO1*, were highly expressed in Cluster 2. These genes are typically upregulated in the ABC subtype due to persistent inflammatory stimulation^17,18^, indicating that Cluster 2 may be enriched with ABC subtype samples. Such identification of distinct DLBCL subtypes based on molecular stratification and immune cell infiltration patterns offers a promising avenue for personalized treatment strategies.

Systematic CNV assessment revealed directional coordination across 24 FRDEGs wherein amplifications selectively targeted ferroptosis drivers (e.g., *ALOX15*,* IFNG*; lipid peroxidation mediators^19^ while deletions affected suppressors (e.g., *CDKN2A*,* SLC40A1*; antioxidant regulators^20^, collectively enhancing ferroptosis susceptibility in DLBCL subtypes. Integrated with prognostic heterogeneity, the CNV directionality could modulate tumor aggressiveness, supported by the association of *CDKN2A* loss with adverse outcomes in carcinomas^21^. Notably, the relevance of *CDKN2A* deletion to Cluster 1 warrants further validation to elucidate its impact on DLBCL progression.

This study revealed that *BTG2* exhibits the highest mutation frequency (28%) in DLBCL, concurrent with substantial copy number variations among FRDEGs. These genomic alterations suggest the potential involvement of BTG2 in DLBCL pathogenesis through aberrant molecular regulation^22,23^. Extensive evidence supports BTG2’s tumor suppressor function via downregulation of SLC7A11 (a critical ferroptosis mediator) thereby modulating cellular susceptibility to oxidative stress^24–26^. However, whether BTG2 directly regulates ferroptosis in DLBCL remains to be experimentally verified.

To identify key regulatory genes, we constructed a PPI network of 24 FRDEGs using the STRING database. Hub genes were prioritized via the DMNC algorithm in CytoHubba, while selecting 15 candidates based on degree centrality and functional weighting. Subsequent dimensionality reduction using LASSO regression with ten-fold cross-validation (λ = 0.05) refined the gene set to three genes with the strongest prognostic association: *CDKN1A*,* KLF2*, and *IFNG*. This multi-tiered strategy ensures identified key genes harbor substantial biological implications and robust clinical relevance. Subsequently, we developed a prognostic risk model based on *CDKN1A*,* KLF2*, and *IFNG* expression that significantly stratifies DLBCL patients into distinct risk groups, thereby potentially informing personalized treatment strategies. Both TCGA-DLBCL and GSE53786 datasets showed elevated expression of all three genes in high-risk groups.

Given the inherent challenges of acquiring clinical samples due to ethical considerations and resource limitations, we included samples from 5 DLBCL patients and 5 healthy controls for preliminary validation. This targeted cohort size, while limited, was selected to provide feasibility for an initial evaluation of expression trends for our TCGA- and GSE53786-derived pivotal genes (*CDKN1A*,* KLF2*,* IFNG*) in a clinical setting. Consistent with our hypothesis, bone marrow immunohistochemistry demonstrated increased CDKN1A/KLF2/IFNG protein in DLBCL-infiltrated marrow. However, peripheral blood qRT-PCR identified *KLF2* as the only transcript significantly upregulated in DLBCL. This RNA-protein discordance may reflect bone marrow-specific post-transcriptional regulatory mechanisms or technical constraints from limited sample sizes. While these initial results provide valuable clinical corroboration for the bioinformatics findings, we acknowledge that future studies with expanded cohort sizes are essential to enhance statistical power and validate the broader applicability of these observations.

CDKN1A exhibits context-dependent duality, functioning as a tumor suppressor through cell cycle arrest^27^ and as an oncogene via pro-survival signaling^28^ in specific malignancies. Our analysis revealed increased CDKN1A expression in DLBCL than controls, suggesting a distinctive regulatory paradigm in lymphoid malignancies. Mechanistically, CDKN1A plays a crucial role in in ferroptosis regulation, such as attenuating ferroptosis induced by hyperoxaluria in renal tubular epithelial cells^29^. KLF2, a multifunctional transcription factor, displays cancer-driving activity specific to certain cell lineages^30^. Consistently, we observed significantly increased KLF2 levels in DLBCL. Moreover, KLF2 has been shown to act as a protective agent by inhibiting ferroptosis through pathways such as SIRT1/GPX4 signaling^31^. IFNG, a crucial cytokine for macrophage activation and antigen presentation facilitation^32^, defines a distinct subtype of hematologic malignancies characterized by immune infiltration and poor chemotherapy outcomes^33^.Beyond its established immunoregulatory roles, IFNG also regulates ferroptosis in cancer cells through inhibition of glutathione synthesis^34,35^, revealing a targetable mechanism for cancer immunotherapy. Collectively, these targets coordinate a microenvironment-modulated iron-immunity axis that differentially governs ferroptosis susceptibility across DLBCL subtypes, providing a mechanistic foundation for subtype-specific therapeutic development.

Epigenetic silencing and reactivation of specific genes represent promising therapeutic targets in high-risk DLBCL. Our study identified significant locus-specific correlations between DNA methylation and expression of *CDKN1A*,* KLF2*, and *IFNG*. These methylation-regulated transcriptional networks functionally augment our *CDKN1A/KLF2/IFNG*-integrated risk stratification framework, enhancing prognostic precision in high-risk DLBCL. Furthermore, focusing on therapeutic vulnerabilities linked to epigenetic modifications can enhance treatment strategies for high-risk DLBCL. Critically, such epigenetic regulation further links to drug sensitivity profiles^36^. Combining traditional chemotherapies with agents that target epigenetic modifiers may enhance treatment efficacy, showcasing a promising avenue for future clinical applications in high-risk DLBCL cohorts^37^.

Our drug sensitivity analysis revealed distinct therapeutic vulnerabilities between molecularly stratified DLBCL subgroups, thereby unlocking clinically actionable strategies. High-risk group sensitizing agents (e.g., Ebelin, Bleomycin, Nilotinib) may benefit patients with chemoresistant or relapsed/refractory DLBCL. For instance, Bleomycin, a component of the R-CHOP regimen, remained effective in high-risk subgroups, which indicated the model’s value in identifying chemotherapy-sensitive populations. While R-CHOP faces resistance challenges, research confirms that ferroptosis induction by rituximab enhances its anti-tumor effect^38^. The enrichment of JNK inhibitors (JNK.9 L) and proteasome inhibitors (Z.LLNle.CHO) in the high-risk group suggests aberrant stress pathway activation and proteolytic dysfunction, which may facilitate therapy evasion by suppressing ferroptosis^39^. This knowledge is crucial for developing targeted therapies to overcome these resistance mechanisms. NSC87877 (paclitaxel) demonstrated significant efficacy against high-risk DLBCL, which is consistant with its role in microtubule stabilization^40^, epigenetic modulation via HDACi synergy^41^, and remodeling tumor microenvironment to in CD47-targeted therapy^42^. Significantly, Paclitaxel’s therapeutic impact hinges crucially on ferroptosis, where activating ferroptosis via the CREB1/GPX4 axis increases paclitaxel sensitivity^43^. Paclitaxel-resistant breast cancer cells demonstrate diminished ferroptosis vulnerability, while integration of ferroptosis in treatment regimens can potentially re-sensitize resistant cancer cells to paclitaxel^44^. Therefore, combining ferroptosis induction with high-risk sensitizing agents (e.g., Bleomycin, NSC87877/paclitaxel) hold translational potential for R-CHOP-resistant malignancies. Conversely, low-risk group sensitizing agents include targeted therapies (BIBW2992 blocking EGFR/Her2) and metabolic modulators (Metformin), potentially offering alternative regimens to reduce chemotherapy toxicity in this cohort. Low-risk patients exhibited enhanced sensitivity to EGFR/PI3K-axis inhibitors like BIBW2992 (afatinib), aligning with aberrant PI3K/AKT/mTOR activation in lymphoid malignancies and supporting EGFR-targeted monotherapy to reduce chemotherapy burden^45^. Furthermore, AMG-706 (motesanib)—a potent multi-kinase inhibitor—demonstrated unprecedented anti-lymphoma efficacy, thereby extending its established anti-angiogenic activity in solid tumors^46^ to lymphoid malignancies. Its multi-kinase inhibition profile (targeting VEGFR/RET) addresses unmet needs in relapsed/refractory DLBCL, particularly when combined with metabolic modulators like metformin in low-risk subgroups. Collectively, molecularly stratified DLBCL reveals distinct therapeutic vulnerabilities: high-risk patients (e.g., ABC subtype) require DNA damage/kinase-stress targeting for aggressive/relapse-prone disease, whereas low-risk cases (e.g., GCB subtype) respond to growth factor receptor blockade and metabolic modulation in driver-dependent malignancies. In light of the FRGs characteristics identified in this study, future research could explore the potential of combining these drugs with ferroptosis inducers, such as RSL3.

While this study provides novel mechanistic insights into DLBCL pathogenesis through systematic integration of multi-omics bioinformatics approaches and clinical data, particularly implicating ferroptosis regulation, several inherent limitations should be acknowledged. First, the experimental data remain inadequate to comprehensively validate the observed associations, and the restricted cohort size may limit statistical generalizability, potentially affecting extrapolation to broader populations. Additionally, the high heterogeneity of DLBCL subtypes and the exclusion of certain datasets (e.g., GSE125966 due to drug treatment-related confounding) may introduce selection bias. The small sample size for experimental validation (e.g., 5 DLBCL cases and 5 controls) further underscores the need for cautious interpretation. These constraints highlight the importance of future studies involving expanded cohorts, multi-center collaborations, and additional experimental verification to strengthen the clinical relevance and robustness of our findings. Nevertheless, our work offers a foundational framework for further exploration of ferroptosis-related mechanisms in DLBCL.

In conclusion, this study emphasizes the pivotal role of ferroptosis in the pathogenesis of DLBCL, revealing its potential as a promising biomarker and therapeutic target. The prognostic risk model we developed, featuring CDKN1A, KLF2, and IFNG, shows promise in predicting patient outcomes and facilitating personalized treatment approaches. Future research should prioritize the experimental validation of identified biomarkers and the assessment of their clinical implications in larger cohorts. This would ultimately enable the development of innovative diagnostic and treatment strategies in addressing the challenges posed by this aggressive malignancy.

### Methods

### Patient sample Preparation

Peripheral blood samples from newly diagnosed patients with DLBCL were collected at Henan Provincial People’s Hospital (Zhengzhou, China) for RNA quantification. Diagnosis confirmation followed the 2022 World Health Organization Classification of Haematolymphoid Tumours, requiring concordant histopathological and immunophenotypic assessments (CD20^+^/CD79a^+^ with > 70% Ki-67 proliferation index). Normal samples were obtained from individuals with no history of hematological disorders or systemically impactful diseases (e.g., malignancies, autoimmune diseases, or organ dysfunction per NIH protocol #09-C-0243). Mononuclear cell isolation utilized Ficoll Hypaque Solution (Haoyang Institute of Biotechnology, Tianjin, China) through standardized density gradient centrifugation. This biomaterial collection protocol (#2024 − 141) received expedited approval from the hospital’s Institutional Review Board and strictly adhered to the ethical principles outlined in the Declaration of Helsinki (2013 revision) and China’s Good Clinical Practice guidelines. Bone marrow biopsy specimens from 5 DLBCL patients with bone marrow involvement and 5 age-matched healthy donors were collected as paraffin-embedded tissues for immunohistochemical analysis.

### RNA extraction and quantitative analysis

Total RNA was extracted using the TRIzol reagent (Servicebio, Wuhan, China) per manufacturer instructions, and RNA concentrations were measured using a NanoDrop instrument (Thermo Fisher Scientific, MA, USA). cDNA templates were synthesized using the cDNA Synthesis SuperMix (Kermey, Zhengzhou, China). qRT-PCR reactions were performed in a StepOne™ real-time PCR system (Thermo Fisher, Waltham, USA) using Universal SYBR Green Supermix (Kermey, Zhengzhou, China), per the manufacturer instructions. *GAPDH* served as an internal reference gene. Specific primer sequences used in this study were listed in Additional Table S4.

### Immunohistochemical staining

Bone marrow biopsy specimens were fixed in 10% neutral buffered formalin and embedded in paraffin blocks. Immunohistochemical staining was commercially performed by Servicebio Biotechnology Co., Ltd (Wuhan, China). Consecutive 4-µm tissue sections were stained using the following primary antibodies under standardized conditions: Anti-CDKN1A/p21 (1:300; Cat# CY5088; Abways Technology, China), Anti-KLF2 (1:300; Cat# ER1911-98; HUABIO, Hangzhou, China), and Anti-IFNG (1:200; Cat# 52522; GenuIN Biotech, Hefei, China). Sections from DLBCL patients and normal bone marrow controls were processed simultaneously with appropriate positive/negative controls.

### Data acquisition and DLBCL subtype construction

TCGA-DLBCL database (*n* = 48) was retrieved using the R package TCGAbiolink^47^. Transcriptomic data underwent TPM normalization prior to differential expression analysis. For survival modeling, a curated subset with complete clinical annotations (*n* = 42) was utilized for survival modeling. DLBCL dataset GSE53786 (Affymetrix HG-U133 Plus 2.0 Array, GPL570) was extracted using the R package GEOquery from the Gene Expression Omnibus (GEO) database (https://www.ncbi.nlm.nih.gov/geo/query/acc.cgi?acc=GSE53786). This cohort comprises 119 clinically annotated DLBC samples, and all were utilized for subsequent validation analyses.

To assess the role of ferroptosis in DLBCL, we retrieved 369 genes identified as drivers, 348 genes recognized as suppressors, and 11 genes serving as markers of ferroptosis from the FerrDb database^48^. A list of the FRGs were summarized in Additional Table S5. To construct the DLBCL subtypes, we used a consensus clustering approach through the R package ConsensusClusterPlus^49^. We established 10 clusters and executed 100 resampling iterations. During each iteration, 80% of the entire sample set was randomly selected. The “pam” clustering algorithm was employed, while utilizing the “minkowski” distance metric for the analysis.

### Analysis of gene expression changes linked to ferroptosis in DLBCL

Gene expression differences were examined using the R package DESeq2^50^. DEGs were determined using the selection criteria of |log_2_FC| > 0.5 and *p* < 0.05. Specifically, genes exhibiting log_2_FC > 0.5 and *p* < 0.05 were classified as upregulated DEGs, whereas genes demonstrating log_2_FC < −0.5 and *p* < 0.05 were categorized as downregulated DEGs. The results of the differential expression analysis were represented by a volcano plot created using the R package ggplot2. To determine the FRDEGs linked to DLBCL, we initially obtained all DEGs from the TCGA-DLBCL dataset meeting the threshold criteria of |log_2_FC| > 0.5 and *p*< 0.05 through differential expression analysis. These DEGs were subsequently intersected with FRGs, and this was visualized using a Venn diagram. The resultant overlapping genes were designated as FRDEGs. Meanwhile, the chromosomal localization map was created using the R package RCircos^51^..

### Genomic landscape characterization in DLBCL

To analyze SMs in the DLBCL samples, we used the “Masked SM” data from TCGA. Data were processed and visualized using the maftools R package^52^. For CNV analysis, we selected the “Masked Copy Number Segment” data from TCGA for DLBCL samples. CNV segments were downloaded, processed, and analyzed using GISTIC2.0^53^, with all parameters set to default settings.

### Functional enrichment profiling of molecular pathways

Functional enrichment profiling was conducted to elucidate the biological significance of FRDEGs using the clusterProfiler package (v4.0.5) in R^54^. Subsequently, GO analysis and KEGG were performed. The criteria for selecting significant entries were set as adjusted p (adj.p) < 0.05 and false discovery rate (FDR, q-value) < 0.25, with p correction performed using the Benjamini–Hochberg method.

### Analysis of gene set enrichment and variability

GSEA was conducted using the R package clusterProfiler with genes ranked by log_2_FC values; parameters were as follows: seed = 2,024, permutations = 1,000, min gene set size = 10, and max gene set size = 500. Gene sets from the Molecular Signatures Database (MSigDB, c2.all.v2023.2. Hs.symbols.gmt)^55^ were used, and significant gene sets were selected based on adj.*p* < 0.05 and FDR < 0.25, with p correction using the Benjamini–Hochberg method. GSVA was performed on TCGA-DLBCL dataset using gene sets from MSigDB. Functional enrichment differences between Clusters 1 and 2 were calculated, with significant gene sets selected with an adj.*p* < 0.05, and corrected using the Benjamini–Hochberg method.

### CIBERSORT immune infiltration analysis

The CIBERSORT algorithm employs linear support vector regression to deconvolve transcriptomic expression matrices, enabling quantitative estimation of immune cell composition and abundance within heterogeneous tissue samples. We implemented CIBERSORT with the LM22 signature gene matrix. Immune cell fractions with enrichment scores > 0 were retained, generating an immune infiltration matrix visualized through stacked bar plots. Comparative analysis of LM22 immune cell infiltration between Cluster 1 and Cluster 2 subgroups was subsequently performed using ggplot2 (v3.4.2).

### Tumor microenvironment and immune feature analysis

To evaluate the impact of tumor microenvironment cells and immune/stromal cell infiltration on prognosis, we used the ESTIMATE package in R^56^ to analyze TCGA-DLBCL dataset. This analysis provided immune scores, stromal scores, ESTIMATE scores, and estimates of tumor purity, which were visualized using ggplot2 to compare Clusters 1 and 2. We also obtained tumor mutation burden and microsatellite instability (MSI) data from cBioPortal (https://www.cbioportal.org/) and calculated the differences between the two clusters. In addition, we used TIDE website (http://tide.dfci.harvard.edu/login/) to perform TIDE immune scoring, which predicts treatment response and survival effects. The TIDE scores were analyzed and compared between Clusters 1 and 2 within TCGA-DLBCL dataset.

### Construction of protein interaction map and identification of hub genes

A PPI network was constructed using STRING database to identify interactions among FRDEGs. The minimum interaction threshold was set at 0.150 (low confidence). Genes exhibiting interaction connections within the PPI network were selected for subsequent analysis. The CytoHubba plugin^57^ in Cytoscape^58^ was used to calculate DMNC scores for the FRDEGs. The hub genes were determined to be the top 15 genes that exhibited the highest scores in the DMNC analysis. The Gene Set Cancer Analysis platform^59^ was employed to evaluate the expression patterns of the hub genes across different cancer types.

### LASSO regression analysis and key gene validation

LASSO regression analysis was performed using the glmnet package in R^60^ with seed = 2,024 and family = “cox,” including ten-fold cross-validation. Hub genes were identified as key genes and visualized using prognostic risk plots and coefficient trajectory plots. The LASSO risk score (Risk Score) was calculated as follows:

$$\:Risk\:Score=\sum\:_{i}coefficient\:\left(gene\:i\right)\ast\:expression\:\left(gene\:i\right)$$

A multivariate Cox proportional hazards regression analysis was conducted using the risk score and clinical data. The DLBCL samples were categorized into high- and low-risk groups based on the median risk score. Group comparisons and risk factor plots were generated for TCGA-DLBCL and GSE53786 datasets. Time-dependent ROC curves were generated using the R package timeROC to assess the predictive accuracy for 1-, 3-, and 5-year survival outcomes. Area under the curve (AUC) values were computed to assess model accuracy, with higher AUC values (closer to 1) indicating better performance.

### DNA methylation analysis

The HM450K array data from TCGA was utilized, with the “Methylation Beta Value” data being downloaded to reflect methylation levels. Quality control and normalization were performed using the ChAMP package^61^. Differentially methylated probes and their values were identified. The RIdeogram package^62^ visualized these values using chromosome ideograms. Heatmaps of beta values for key genes were generated using pheatmap, and correlation heatmaps between key genes and methylation sites were created using Corrplot.

### Drug sensitivity analysis

The feature of cancer drug sensitivity genome was extracted from GDSC database (https://www.cancerrxgene.org/). Utilizing the pRRophetic algorithm^63^, we forecast the diagnostic sensitivity in both high- and low-risk cohorts within TCGA-DLBCL dataset by calculating IC50 values. Results were visualized using group comparison plots.

### Creation of a predictive risk framework for DLBCL

We performed both single-variable and multi-variable Cox proportional hazards regression analyses using the R survival package to construct a predictive risk model for the TCGA-DLBCL dataset. Univariate Cox regression results were visualized using a forest plot showing key gene expression and clinical information. A nomogram was developed employing the “rms” package in R to depict the associations between significant genes and clinical variables within the context of a multivariate Cox regression framework. Calibration plots were created to evaluate the model’s predictive precision by comparing actual and predicted probabilities. DCA was performed with the R package ggDCA to evaluate the clinical applicability of the model based on key gene expression and clinical information^64^.

### Statistical analysis

All data processing and analyses were conducted using the R software (version 4.2.1). Continuous variables are expressed as the mean ± standard deviation. The Wilcoxon rank-sum test was applied for comparisons between two distinct groups. In the absence of specific indications, Spearman correlation analysis was implemented to evaluate the correlation coefficients among various molecules, with *p* < 0.05 denoting statistical significance.

## Supplementary Information

Below is the link to the electronic supplementary material.

Supplementary Material 1

Supplementary Material 2

## Data Availability

The DLBCL RNA-seq and clinical data that support the findings of this study are openly available in TCGA (https://www.cancer.gov/ccg/research/genome-sequencing/tcga) and GEO (https://www.ncbi.nlm.nih.gov/geo/query/acc.cgi? acc=GSE53786). FRGs are publicly available in FerrDb database (http://www.zhounan.org/ferrdb/current/). TMB and MSI data can be retrieved from cBioPortal platform (https://www.cbioportal.org/). PPI network data is openly available in STRING (https://cn.string-db.org/). The data of drug sensitivity in cancer is openly available in GDSC database (https://www.cancerrxgene.org/). The other data supporting the findings of this study are available from the corresponding author on reasonable request.

## Abbreviations

AUV: area under the curve
CNV: copy number variation
DLCBL: diffuse large B-cell lymphoma
DEGs: differentially expressed genes
DMNC: Density of Maximum Neighborhood Component
FDR: false discovery rate
FRDEGs: ferroptosis-related differentially expressed genes
FRGs: ferroptosis-related genes
GDSC: Genomics of Drug Sensitivity in Cancer
GEO: Gene Expression Omnibus
GO: Gene Ontology
GSEA: Gene set enrichment analysis
GSVA: Gene set variation analysis
KEGG: Kyoto Encyclopedia of Genes and Genomes
MSI: microsatellite instability
NK: natural killer
PPI: protein-protein interaction
ROC: receiver operating characteristic
SMs: somatic mutations
TCGA: The Cancer Genome Atlas
TIDE: tumor immune dysfunction and exclusion
TMB: tumor mutation burden
