# Supplementary material for: Ferroptosis-related gene analysis revealing novel biomarkers and therapeutic targets in diffuse large B-cell lymphoma
Source: Sci Rep. 2025 Nov 4;15:38613. doi: 10.1038/s41598-025-22478-1 (PMC12586583; doi:10.1038/s41598-025-22478-1)
Supplement: Supplementary file 2 — Supplementary Material 2 [file 41598_2025_22478_MOESM2_ESM.docx]

**Supplementary files**

**Fig. S1 Consensus Clustering Analysis of DLBCL** (.pdf)

**Fig. S2 CNV and SM Analysis of DLBCL** (.docx)

**Fig. S3. Protein Interaction Networks and prognostic significance of hub genes** (.docx)

**Fig. S4: Time-Dependent ROC Validation of Key Genes in GSE53786** (.docx)

**Fig. S5: Methylation Analysis of Key Genes in TCGA-DLBCL** (.docx)

**Table S1 Results of GO and KEGG Enrichment Analysis of FRDEGs** (.docx)

**Table S2 GSEA Results of TCGA-DLBCL** (.docx)

**Table S3 GSVA Results of TCGA-DLBCL** (.docx)

**Table S4 The primer sequences for qRT-PCR** (.docx)

**Table S5 FRGs catalog from FerrDb database** (.xlsx)

## Supplementary Figure Legends


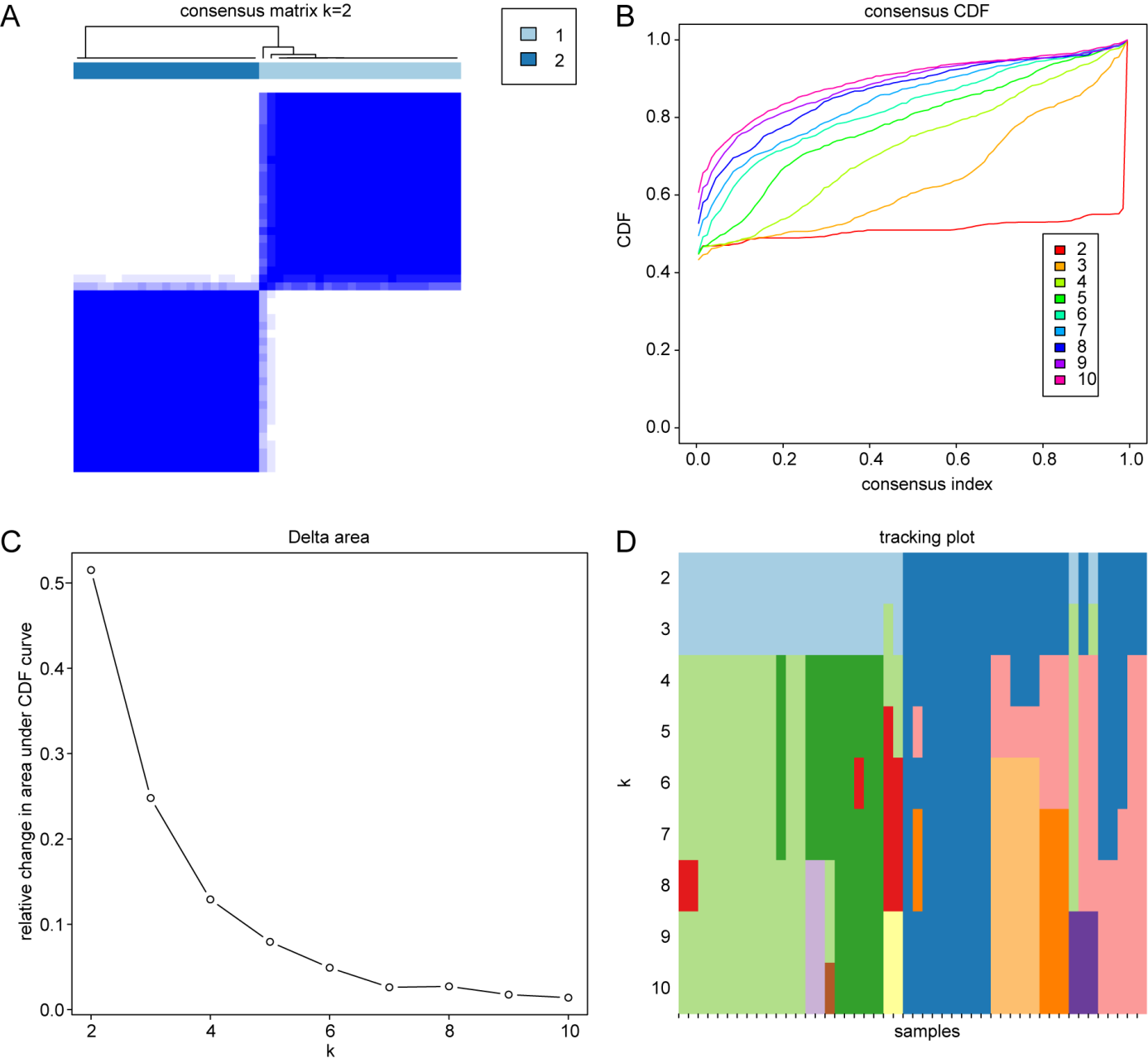


**Figure S1. Consensus Clustering Analysis of DLBCL**

A. Plot of consensus clustering results for DLBCL samples. B–D. (B) Consistency cumulative distribution function (CDF) plot, (C) delta plot, and (D) tracking plot (for consistency clustering analysis). DLBCL, diffuse large B-cell lymphoma.


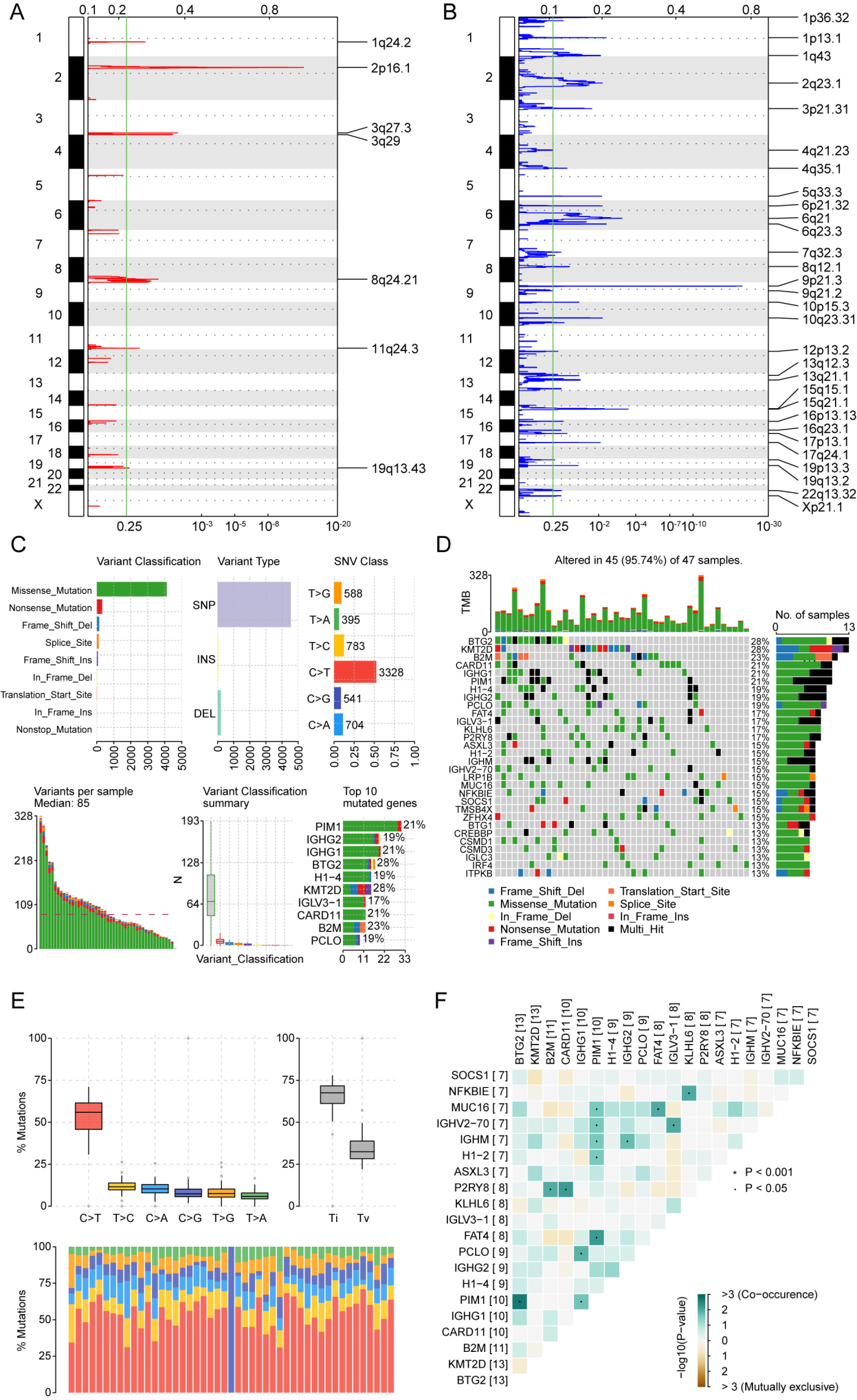


**Figure S2. CNV and SM Analysis of DLBCL**

A–B. GISTIC plot of Amplification (A) and Deletion (B) in TCGA-DLBCL. G scores are shown on the top, and q values are shown on the bottom, showing the locations of significant Amplification and Deletion for each chromosome. The ones to the right of the green vertical line are statistically significant. C. SM presentation in TCGA-DLBCL dataset. The bar chart and box plot show the comparison of different mutation types and modes, respectively. D. Presentation of the Top 30 SMs in the dataset. Colors indicate different mutation types. E. Boxplot and stacked bar chart of mutation types. F. Mutation correlation heatmap of the top 20 genes with mutation frequency. DLBCL, diffuse large B-cell lymphoma; CNV, Copy Number Variations; SM, somatic mutation; TCGA, The Cancer Genome Atlas.

**
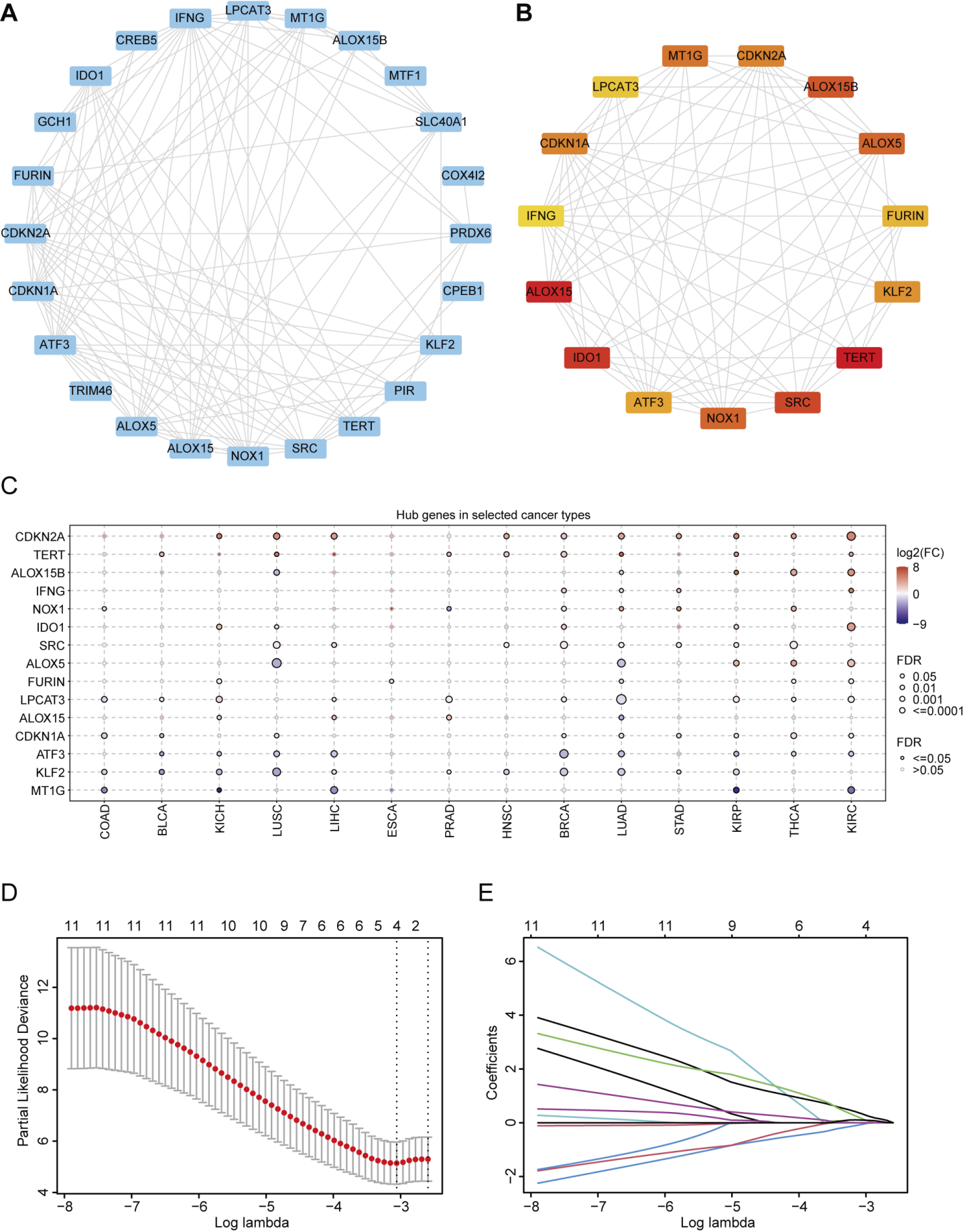
**

**Figure S3. Protein Interaction Networks and prognostic significance of hub genes**

A. PPI network of FRDEGs calculated using the STRING database. Nodes represent proteins, and edges represent interactions. B. PPI network of hub genes identified using the DMNC algorithm in the CytoHubba plugin. The color intensity reflects the ranking of each gene according to the DMNC algorithm, with deeper red indicating a higher rank. C. Heatmap illustrating the differential expression of hub genes across various cancer types using the GSCA website. Red indicates upregulation relative to the normal group, while blue indicates downregulation. The circle size represents the False Discovery Rate (FDR). D. Prognostic risk model generated by the LASSO regression analysis for identifying significant hub genes. The x-axis represents the log (λ) values, and the y-axis shows the coefficients of the selected variables. The vertical dashed line corresponds to the λ value selected by cross-validation. E. Variable trajectory plot from the LASSO regression model. Each line represents the path of the coefficient of a variable as λ increases. Variables that remain non-zero at higher λ values are considered more significant for the prognostic model. GSCA, Gene Set Cancer Analysis.


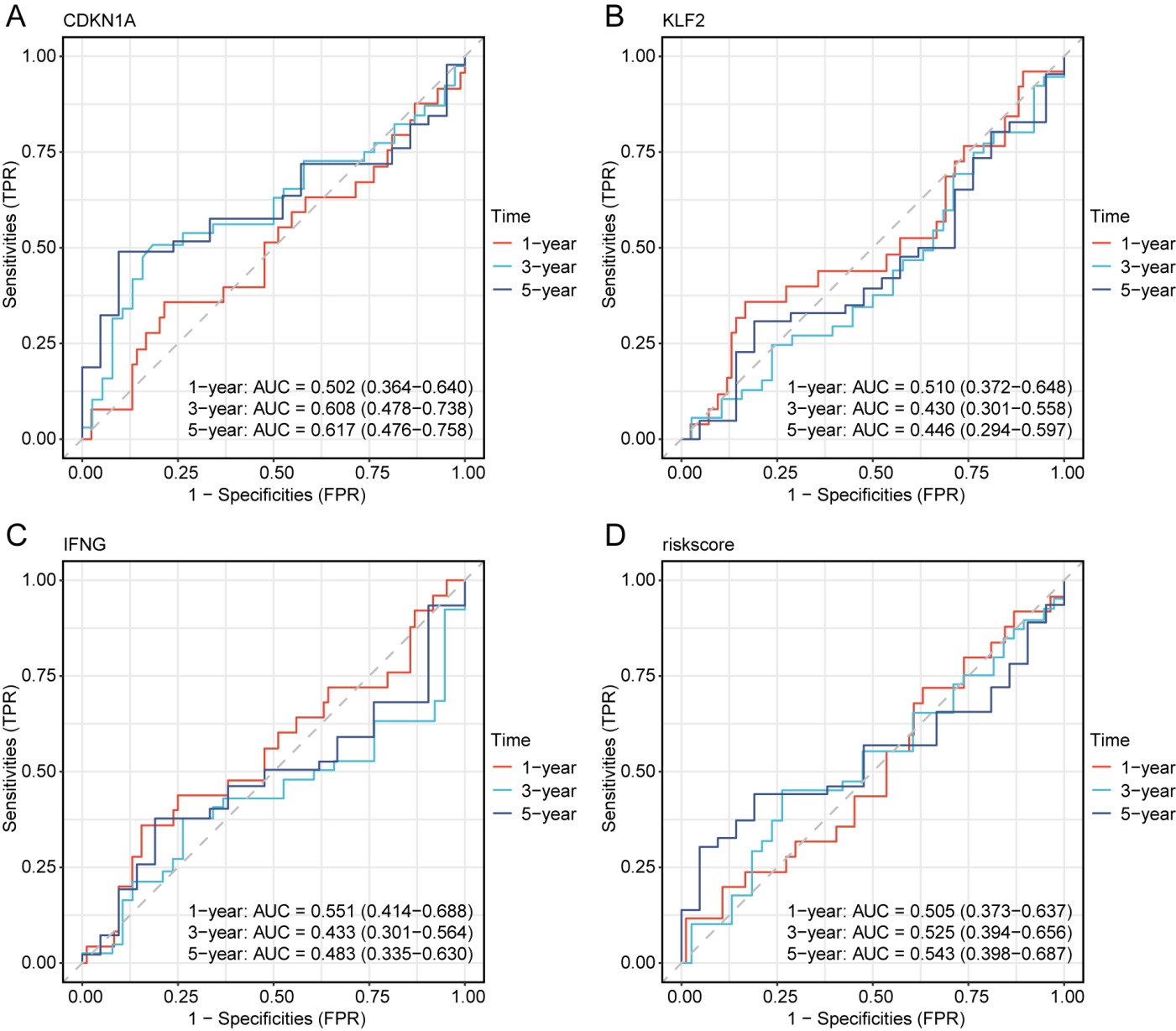


**Figure S4. Time-Dependent ROC Validation of Key Genes in GSE53786**

Time-dependent ROC curves for the key genes *CDKN1A* (A), *KLF2* (B), and *IFNG* (C) and the risk score (D) in the GSE53786 dataset at 1, 3, and 5 years. The x-axis represents the false positive rate (FPR), and the y-axis represents the true positive rate (TPR). The AUC values are provided for each time point. ROC, receiver operating characteristic; AUC, area under the curve.


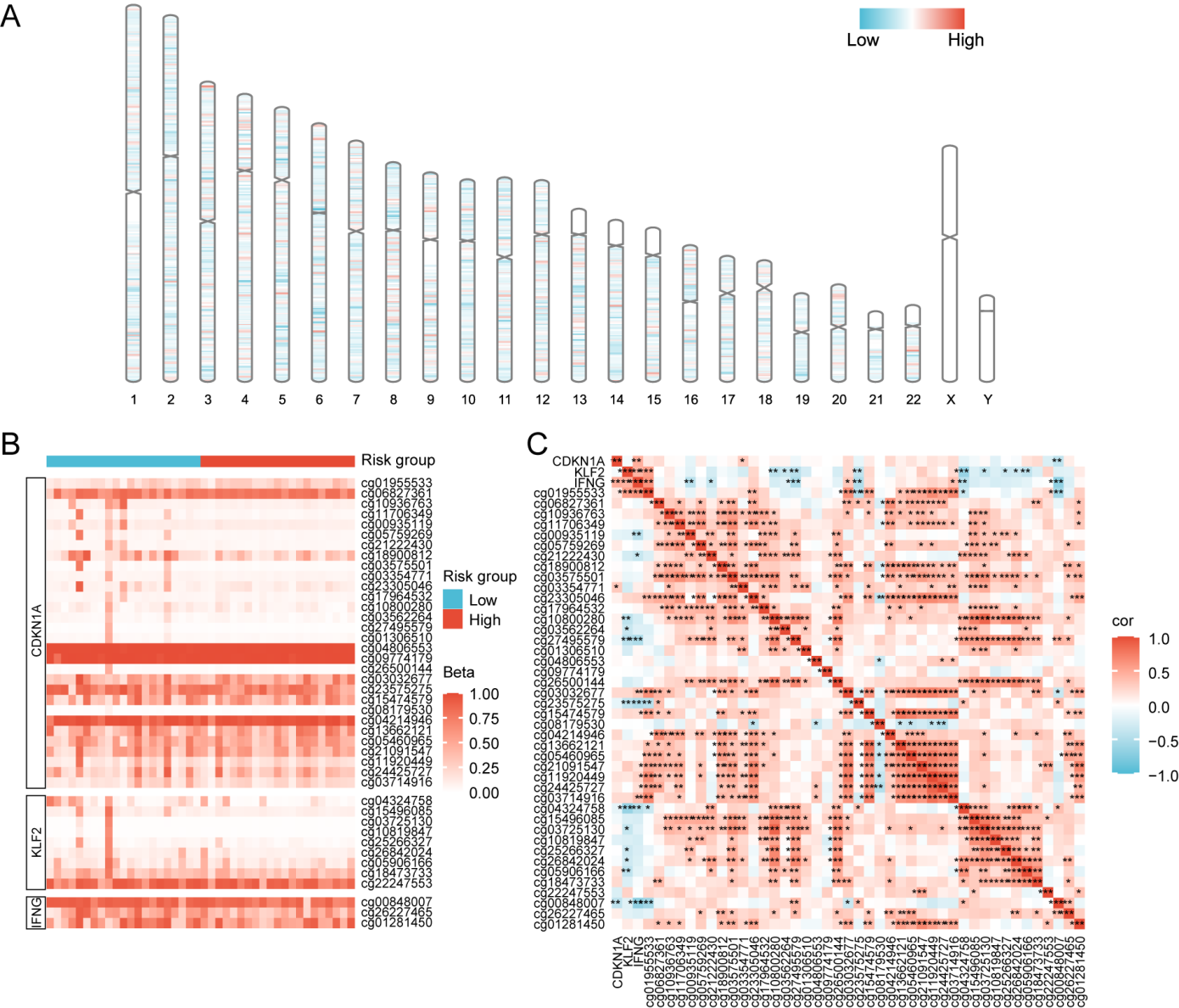


**Figure S5. Methylation Analysis of Key Genes in TCGA-DLBCL**

A. Chromosomal localization of differential methylation sites in TCGA-DLBCL dataset. Each short line represents a methylation site, with red and blue lines indicating positive and negative deltaBeta values, respectively. Color intensity reflects the magnitude of the deltaBeta value. B. Heatmap of the methylation levels (Beta values) of 42 methylation sites for three key genes in the high-risk (red samples) and low-risk (blue samples) groups. Color intensity indicates the Beta value. C. Correlation heatmap showing the correlations between the expression of three key genes and the Beta values of the methylation levels at 42 sites. Red and blue colors indicate positive and negative correlations, respectively. Color intensity reflects the absolute value of the correlation coefficient. * p < 0.05, ** p < 0.01; *** p < 0.001. TCGA, The Cancer Genome Atlas; DLBCL, diffuse large B-cell lymphoma.

**
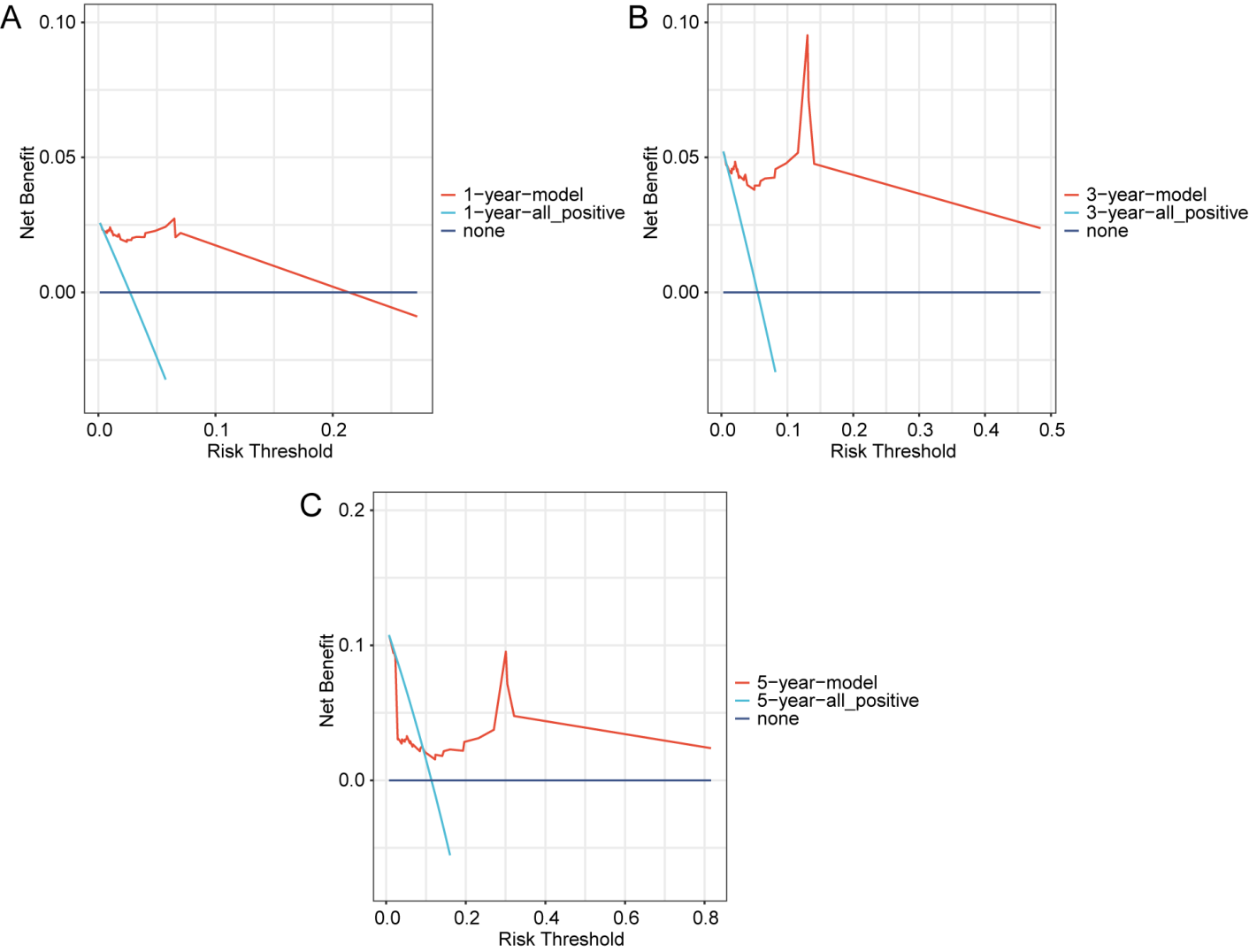
**

**Figure S6. Decision Curve Analysis (DCA) of DLBCL**

(A) DCA for 1-year survival in TCGA-DLBCL dataset. (B) DCA for 3-year survival in TCGA-DLBCL dataset. (C) DCA for 5-year survival in TCGA-DLBCL dataset. TCGA, The Cancer Genome Atlas; DLBCL, diffuse large B-cell lymphoma.

## 2. Supplementary Tables

**Table S1 Results of GO and KEGG Enrichment Analysis of FRDEGs**

| ONTOLOGY | ID | Description | GeneRatio | p | p.adjust | q-value | Count |
| --- | --- | --- | --- | --- | --- | --- | --- |
| BP | GO:0019372 | Lipoxygenase pathway | 3/24 | 2.95E-07 | 3.91E-04 | 2.26E-04 | 3 |
| BP | GO:0050727 | Regulation of inflammatory response | 7/24 | 5.76E-07 | 3.91E-04 | 2.26E-04 | 7 |
| BP | GO:0006979 | Response to oxidative stress | 7/24 | 7.91E-07 | 3.91E-04 | 2.26E-04 | 7 |
| BP | GO:0090399 | Replicative senescence | 3/24 | 1.21E-06 | 4.48E-04 | 2.59E-04 | 3 |
| BP | GO:0043651 | Linoleic acid metabolic process | 3/24 | 2.36E-06 | 5.60E-04 | 3.24E-04 | 3 |
| MF | GO:0016702 | Oxidoreductase activity, acting on single donors with incorporation of molecular oxygen, incorporation of two atoms of oxygen | 5/24 | 1.00E-10 | 9.46E-09 | 6.13E-09 | 5 |
| MF | GO:0016701 | Oxidoreductase activity, acting on single donors with incorporation of molecular oxygen | 5/24 | 1.25E-10 | 9.46E-09 | 6.13E-09 | 5 |
| MF | GO:0051213 | Dioxygenase activity | 5/24 | 1.16E-07 | 5.82E-06 | 3.77E-06 | 5 |
| MF | GO:0004861 | Cyclin-dependent protein serine/threonine kinase inhibitor activity | 2/24 | 1.06E-04 | 4.02E-03 | 2.60E-03 | 2 |
| MF | GO:0030291 | Protein serine/threonine kinase inhibitor activity | 2/24 | 8.37E-04 | 2.51E-02 | 1.63E-02 | 2 |
| KEGG | hsa04216 | Ferroptosis | 3/20 | 1.06E-04 | 7.70E-03 | 6.03E-03 | 3 |
| KEGG | hsa05219 | Bladder cancer | 3/20 | 1.06E-04 | 7.70E-03 | 6.03E-03 | 3 |
| KEGG | hsa05418 | Fluid shear stress and atherosclerosis | 4/20 | 2.52E-04 | 1.22E-02 | 9.55E-03 | 4 |
| KEGG | hsa00590 | Arachidonic acid metabolism | 3/20 | 3.48E-04 | 1.26E-02 | 9.89E-03 | 3 |
| KEGG | hsa05203 | Viral carcinogenesis | 4/20 | 1.08E-03 | 2.81E-02 | 2.20E-02 | 4 |

GO, Gene Ontology; KEGG, Kyoto Encyclopedia of Genes and Genomes; FRDEGs, Ferroptosis-Related Differentially Expressed Genes; BP, Biological Process; MF, Molecular Function.

**Table S2 GSEA Results of TCGA-DLBCL**

| Description | Set size | Enrichment score | NES | p | p.adjust | q-value |
| --- | --- | --- | --- | --- | --- | --- |
| REACTOME EUKARYOTIC TRANSLATION INITIATION | 99 | -0.620 | -2.505 | 1.00E-10 | 2.71E-08 | 2.27E-08 |
| ALTEMEIER RESPONSE TO LPS WITH MECHANICAL VENTILATION | 112 | 0.726 | 2.498 | 1.00E-10 | 2.71E-08 | 2.27E-08 |
| VERHAAK AML WITH NPM1 MUTATED UP | 153 | 0.673 | 2.417 | 1.00E-10 | 2.71E-08 | 2.27E-08 |
| BLANCO MELO COVID-19 SARS COV 2 POS PATIENT LUNG TISSUE UP | 125 | 0.687 | 2.415 | 1.00E-10 | 2.71E-08 | 2.27E-08 |
| REACTOME RRNA PROCESSING | 167 | -0.532 | -2.290 | 1.00E-10 | 2.71E-08 | 2.27E-08 |
| BLANCO MELO RESPIRATORY SYNCYTIAL VIRUS INFECTION A594 CELLS UP | 241 | 0.612 | 2.285 | 1.00E-10 | 2.71E-08 | 2.27E-08 |
| BOSCO TH1 CYTOTOXIC MODULE | 99 | 0.674 | 2.282 | 1.00E-10 | 2.71E-08 | 2.27E-08 |
| BLANCO MELO HUMAN PARAINFLUENZA VIRUS 3 INFECTION A594 CELLS UP | 177 | 0.628 | 2.281 | 1.00E-10 | 2.71E-08 | 2.27E-08 |
| REACTOME TRANSLATION | 247 | -0.507 | -2.232 | 1.00E-10 | 2.71E-08 | 2.27E-08 |
| BLANCO MELO COVID19 SARS COV 2 INFECTION CALU3 CELLS UP | 280 | 0.585 | 2.215 | 1.00E-10 | 2.71E-08 | 2.27E-08 |
| TAKEDA TARGETS OF NUP98 HOXA9 FUSION 3D UP | 164 | 0.594 | 2.145 | 1.00E-10 | 2.71E-08 | 2.27E-08 |
| ZHANG'S RESPONSE TO IKK INHIBITOR AND TNF UP | 196 | 0.585 | 2.141 | 1.00E-10 | 2.71E-08 | 2.27E-08 |
| KEGG CYTOKINE RECEPTOR INTERACTION | 203 | 0.582 | 2.138 | 1.00E-10 | 2.71E-08 | 2.27E-08 |
| FULCHER INFLAMMATORY RESPONSE LECTIN VS LPS DN | 396 | 0.553 | 2.138 | 1.00E-10 | 2.71E-08 | 2.27E-08 |
| RUTELLA RESPONSE TO HGF VS CSF2RB AND IL4 UP | 382 | 0.553 | 2.128 | 1.00E-10 | 2.71E-08 | 2.27E-08 |
| RUTELLA RESPONSE TO HGF UP | 397 | 0.540 | 2.086 | 1.00E-10 | 2.71E-08 | 2.27E-08 |
| RUTELLA RESPONSE TO CSF2RB AND IL4 DN | 296 | 0.544 | 2.065 | 1.00E-10 | 2.71E-08 | 2.27E-08 |
| MCLACHLAN DENTAL CARIES UP | 225 | 0.546 | 2.029 | 1.00E-10 | 2.71E-08 | 2.27E-08 |
| BLANCO MELO BETA INTERFERON TREATED BRONCHIAL EPITHELIAL CELLS UP | 320 | 0.530 | 2.024 | 1.00E-10 | 2.71E-08 | 2.27E-08 |
| HECKER IFNB1 TARGETS | 82 | 0.686 | 2.271 | 1.94E-10 | 5.00E-08 | 4.19E-08 |

TCGA, The Cancer Genome Atlas; DLBCL, Diffuse Large B-Cell Lymphoma; GSEA, Gene Set Enrichment Analysis; NES, Normalized Enrichment Score.

**Table S3 GSVA Results of TCGA-DLBCL**

| ID | logFC | AveExpr | t | P.Value | adj.P.Val | B |
| --- | --- | --- | --- | --- | --- | --- |
| BIOCARTA DICER PATHWAY | 1.17 | -0.031 | 20.51 | 4.25E-26 | 1.35E-22 | 49.02 |
| REACTOME COHESIN LOADING ONTO CHROMATIN | 1.07 | 0.009 | 19.51 | 4.01E-25 | 8.52E-22 | 46.85 |
| REACTOME SYNTHESIS OF PIPS AT THE ER MEMBRANE | 1.04 | 0.004 | 11.70 | 5.76E-16 | 5.55E-14 | 26.13 |
| FINETTI BREAST CANCERS KINOME GRAY | 1.01 | 0.027 | 15.18 | 2.01E-20 | 1.28E-17 | 36.27 |
| MCGOWAN RSP6 TARGETS DN | 1.01 | 0.012 | 9.91 | 2.02E-13 | 7.74E-12 | 20.32 |
| BIOCARTA TERC PATHWAY | 0.98 | -0.016 | 11.75 | 4.85E-16 | 4.93E-14 | 26.31 |
| WP PILOCYTIC ASTROCYTOMA | 0.95 | -0.022 | 10.81 | 1.00E-14 | 6.12E-13 | 23.30 |
| REACTOME SENSING OF DNA DOUBLE-STRAND BREAKS | 0.94 | -0.018 | 11.71 | 5.47E-16 | 5.44E-14 | 26.19 |
| WP SARSCOV2 REPLICATION ORGANELLE FORMATION | 0.94 | -0.004 | 10.18 | 8.06E-14 | 3.53E-12 | 21.23 |
| WP DDX1 AS A REGULATORY COMPONENT OF THE DROSHA MICROPROCESSOR | 0.94 | -0.035 | 10.41 | 3.78E-14 | 1.88E-12 | 21.98 |
| HOLLEMAN VINCRISTINE RESISTANCE ALL DN | -0.80 | -0.005 | -9.72 | 3.82E-13 | 1.31E-11 | 19.69 |
| REACTOME RESPONSE OF EIF2AK4 GCN2 TO AMINO ACID DEFICIENCY | -0.80 | -0.076 | -9.31 | 1.56E-12 | 4.47E-11 | 18.29 |
| BIOCARTA FOSB PATHWAY | -0.81 | -0.039 | -8.80 | 9.32E-12 | 2.12E-10 | 16.51 |
| WP CYTOPLASMIC RIBOSOMAL PROTEINS | -0.81 | -0.119 | -9.38 | 1.22E-12 | 3.58E-11 | 18.53 |
| REACTOME EUKARYOTIC TRANSLATION ELONGATION | -0.83 | -0.093 | -9.03 | 4.12E-12 | 1.03E-10 | 17.32 |
| KEGG RIBOSOME | -0.86 | -0.111 | -9.69 | 4.26E-13 | 1.44E-11 | 19.58 |
| HOLLEMAN VINCRISTINE RESISTANCE B ALL DN | -0.88 | -0.025 | -15.33 | 1.33E-20 | 1.06E-17 | 36.68 |
| NAKAMURA ALVEOLAR EPITHELIUM | -0.89 | -0.164 | -9.15 | 2.70E-12 | 7.14E-11 | 17.74 |
| YANAGISAWA LUNG CANCER RECURRENCE | -0.90 | -0.011 | -10.48 | 3.02E-14 | 1.54E-12 | 22.21 |
| REACTOME SULFIDE OXIDATION TO SULFATE | -0.93 | -0.015 | -9.10 | 3.30E-12 | 8.48E-11 | 17.54 |

TCGA, The Cancer Genome Atlas; DLBCL, Diffuse Large B-Cell Lymphoma; GSVA, Gene Set Variation Analysis.

**Table S4. The primer sequences for qRT-PCR**

| Name | Sequences |
| --- | --- |
| GAPDH-R | TGCACCACCAACTGCTTAGC |
| GAPDH-F | GGCATGGACTGTGGTCATGAG |
| IFNG-F | TCGGTAACTGACTTGAATGTCCA |
| IFNG-R | TCGCTTCCCTGTTTTAGCTGC |
| KLF2-F | TTCGGTCTCTTCGACGACG |
| KLF2-R | TGCGAACTCTTGGTGTAGGTC |
| CDKN1A-F | TGTCCGTCAGAACCCATGC |
| CDKN1A-R | AAAGTCGAAGTTCCATCGCTC |

**Table S5**

File name: Supplementary file 1

File format: .xlsx

Title of data: FRGs catalog from FerrDb database

Description of data: This file contains 369 ferroptosis driver genes, 348 ferroptosis suppressor genes, and 11 ferroptosis biomarker genes curated from the FerrDb database (Version 2.0).
